# Analysis of México’s Narco-War Network (2007–2011)

**DOI:** 10.1371/journal.pone.0126503

**Published:** 2015-05-18

**Authors:** Jesús Espinal-Enríquez, Hernán Larralde

**Affiliations:** 1 National Institute of Genomic Medicine, Periférico Sur 4809. Arenal Tepepan, 14610, Ciudad de México, México; 2 Center for Complexity Sciences. Ciudad Universitaria, Ciudad de México, México; 3 Insituto de Ciencias Físicas. Universidad Nacional Autónoma de México, Apartado Postal 48-3, Cuernavaca, Morelos 62251, México; Universidad Rey Juan Carlos, SPAIN

## Abstract

Since December 2006, more than a thousand cities in México have suffered the effects of the war between several drug cartels, amongst themselves, as well as with Mexican armed forces. Sources are not in agreement about the number of casualties of this war, with reports varying from 30 to 100 thousand dead; the economic and social ravages are impossible to quantify. In this work we analyze the official report of casualties in terms of the location and the date of occurrence of the homicides. We show how the violence, as reflected by the number of casualties, has increased over time and spread across the country. Next, based on the correlations between cities in the changes of the monthly number of casualties attributed to organized crime, we construct a narco-war network where nodes are the affected cities and links represent correlations between them. We find that close geographical distance between violent cities does not imply a strong correlation amongst them. We observe that the dynamics of the conflict has evolved in short-term periods where a small core of violent cities determines the main theatre of the war at each stage. This kind of analysis may also help to describe the emergence and propagation of gang-related violence waves.

## Introduction and Social Context

During the last 8 years, México has been the scenario of a brutal battle between several armed groups involved in drug trafficking, kidnapping, extortion and many other criminal activities, as well as with the Mexican law enforcement units. This battle has turned many cities of the country into ghost towns. The economic losses are incalculable; the human losses are worse. The main participants in this war are The Sinaloa Cartel, the Gulf Cartel, the Zetas Group, the Beltrán-Leyva Cartel, La Familia Michoacana Cartel (FM), the Juárez Cartel, and the Tijuana Cartel, to name but a few; and the Mexican armed forces (both army and navy), the Mexican Federal Police and the State and Municipal Polices.

This narco-war started at the beginning of 2007 when former Mexican president Felipe Calderón launched “Operación Michoacán” and “Operación Baja California” against the main cartels of those states: FM and Tijuana Cartel [1]. These operations resulted in a surge of violence that spread to other states and cities. This violence became more intense in cities which allegedly are important drug production and distribution points or routes to the United States: cities like Juárez, in Chihuahua state; Reynosa, Tamaulipas (both border cities with the US); Culiacán, Sinaloa; and Uruapan, Michoacán (alleged production centers of marihuana and methamphetamine) [2].

**Fig 1:**
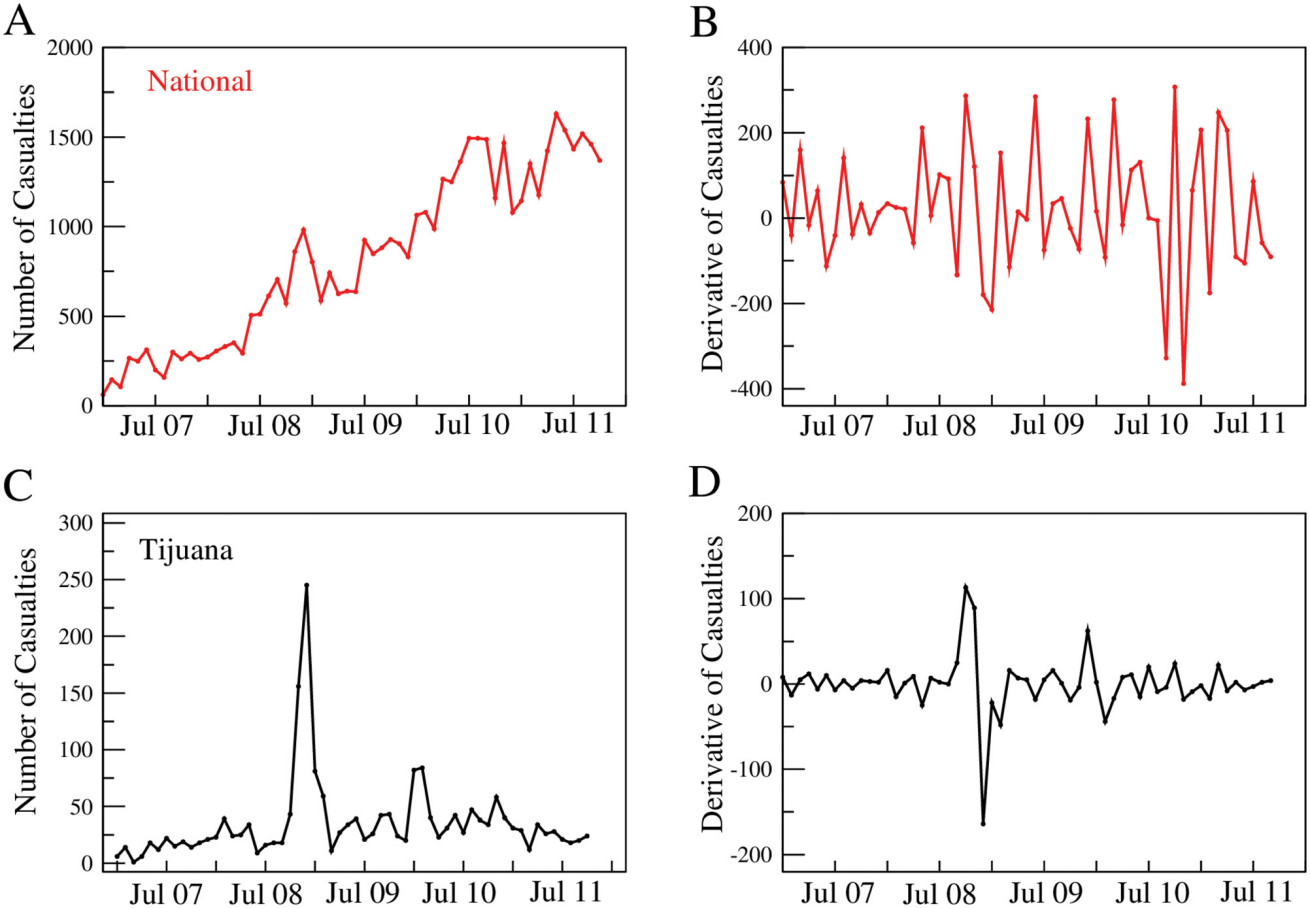
Narco-war-related casualties in México since December 2006. A) Absolute number of casualties in México due to the Narco War. These casualties correspond to people who were either killed in clashes with the Mexican armed forces, or executed during conflicts among drug cartels. The number of deaths during the reported months is clearly increasing. B) Discrete derivative of A. Note that the fluctuations of this quantity also grow in time. C) The number of drug-war-related casualties in the municipality of Tijuana City from December 2006 to September 2011, showing the intermittent nature of the conflict at any given place. D) The discrete derivative of C.

In this work, we present a statistical analysis of the official report of narco-war-related casualties from December 2006 to September 2011 (the original data is no longer available online, the complete data is provided in the supplementary material S1 Dataset, S1 and S2 Tables), in terms of the time and place where the events are presumed to have taken place. First we note that narco-war-related violence is intermittent in time at the local geographical scale (at the smallest geographical scale, México is divided into political entities called *municipios* – municipalities—which is the level at which the data is reported) and inhomogeneously distributed throughout the country at any given time. Furthermore, the aggregate data, the total number of narco-war related casualties in the country, is an overall increasing function of time, as can be seen in Fig 1A.

There have been many attempts to describe, understand, predict or control the dynamics and spread of conflict and gang-related violence using a variety of different approaches: from literature-based approaches [3], reaction–diffusion equations [4], differential equations [5], data-mining-based network inference [6, 7], to combined methods taking into account the geographical and historical aspects of the conflicts [8, 9]. On the other hand, statistical analyses of wars, conflicts and social struggles have been reported previously [10–14]; these works have studied different situations, from civil wars or international conflicts [12, 13], to the dirty war during the military dictatorship period in Argentina [10, 11]. It should be noticed, however, that the available information for many of the studies at the level of gang-related violence often includes the identities of the offenders, the victims and the gangs to which each individual belongs, and even the history of previous conflicts between the gangs. Thus possible motives for conflict, like retaliation for a previous offence, or turf wars, can be hypothesized. In our case, however, the lack of reliable information does not allow us to construct a theoretical framework in which we can recognize the different factions involved in each case. Indeed, it may be that some of the casualties do not even belong to one of the factions in the conflict (for example, some of these criminal groups are also involved in kidnapping and/or illegal immigration, and it is not rare that hostages or illegal immigrants in their hands die or are killed).

Thus, in this work we only use the official available data—the number of drug-war-related deaths each month and where they were found—with no bias from other sources. Of course, the interpretation and analysis of our results could be enriched with further knowledge about the area of influence of the various criminal groups at each stage of the conflict as well as other data which are either non-public or purely speculative. Further, the non-stationary nature of the data at our disposal implies that the statistical tools used to describe stationary processes cannot be used. Also, the data does not (fortunately) consist of distinct realizations, so averaging over realizations is not an option either. Instead, our analysis is based on the construction of a narco-war network for each month, where nodes are the affected regions and links represent a significant correlation in the *change* of the monthly number of casualties reported in the linked regions. Since averaging these correlations in time is not warranted, we focus on which nodes subsist over a certain number of months. We find that the conflict evolves over relatively short-term stages in which a small core of violent cities determines the main theatre of the narco-war at each stage.

As has been pointed out in many previous works, it is very difficult to obtain complete data of all disappearances or murders. For example, in [15], the authors present a comparison between the number of drug-war-related casualties reported by the Mexican Security forces and the Mexican newspaper **Reforma**. They show that the difference between both databases is around 25%. Furthermore, in [16], together with **Reforma** and official data from the government, they compare another Mexican newspaper, **Milenio**, as well as a private consultant company: **Lantia**. Since the criteria to consider a homicide as a result of organized crime vary, these databases are not identical. The criteria used by the government to decide whether a homicide should be attributed to organized crime are:

1. Victim killed by high-caliber or automatic firearms typical of drug cartels (e.g.,.50 caliber)
2. Signs of torture, decapitation, or dismemberment.
3. Body was wrapped in blankets, taped, or gagged.
4. Victim killed at specific locations connected with criminal groups, or in a vehicle.
5. Victim killed by drug cartel while being in jail.
6. Special circumstances (e.g., narco-message; victim alleged drug-cartel member; victim killed after being abducted without asking for ramson; victim ambushed or chased) [16].

In this work we confine ourselves to the official data set made public by the Mexican attorney general’s office (Procuraduría General de la República) in [17] (as mentioned above, the data is presented in S1 Dataset and S1 and S2 Tables), hoping that it includes enough data to yield at least a good qualitative description of the evolution of the narco-war that has been raging in México over the last few years.

## The geographic and time behavior of violence

The growth of violence is shown in Fig 1A, where we plot the *total* number of drug-war-related casualties per month, from December 2006 to September 2011. The graph in Fig 1A shows unambiguously that the total number of casualties essentially grows in time. In contrast, in any given municipality, the number of casualties is very intermittent. In Fig 1C we show the case of Tijuana City, an important trade center between México and the US. Further, we note that even the increase in nationwide violence is non-stationary. This can be observed in Fig 1B and 1D, where we plot the discrete derivative of Fig 1A and 1C, respectively: Fig 1B shows that the fluctuations of the derivative also grow in the course of time. Nonstationarity makes the data analysis difficult, in particular, taking averages over time becomes meaningless because they involve data that is not described by the same statistics.

**Fig 2:**
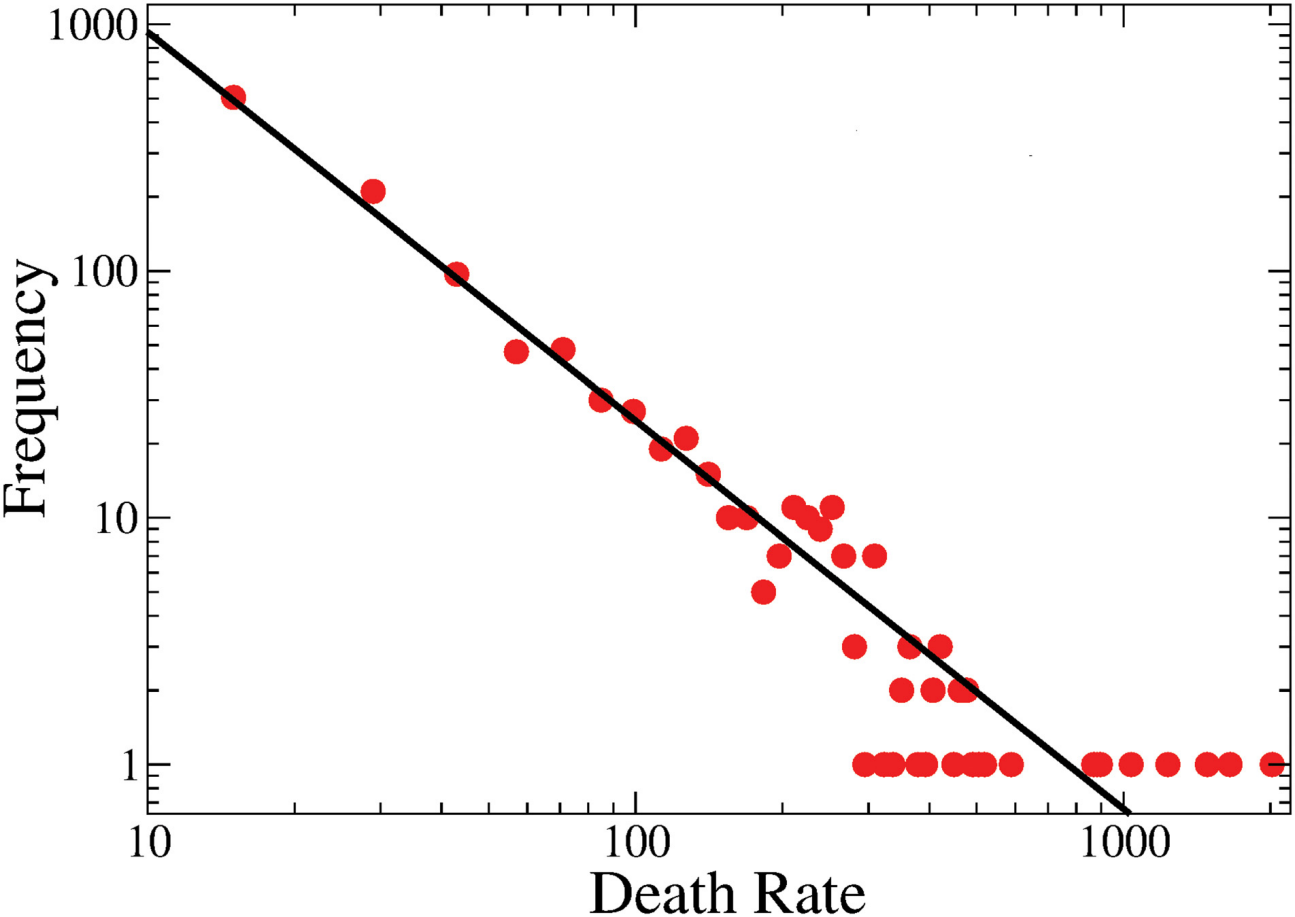
Log–log plot of the cumulative death rate of municipalities per year. Histogram (150 bins) representing the cumulative death rate in all municipalities affected by the organized crime. The data can be fitted well by a power law (black curve). The best fit corresponds to an exponent −1.57±0.025 (*R* ^2^ = 0.9995), the curve corresponds to the equation *y* ≈ (3.48 × 10^4^)*x* ^−1.57^. Thus, some kind of statistical regularity seems to appear despite the non-stationarity of the data. Similar results have been reported in relation to the size of conflicts at least as far back as 1941 [12, 13].

Since the places in which the war is being fought change in time, the number of casualties as time goes by in any given region is a highly irregular intermittent signal, from which few or no conclusions may be drawn (see Fig 1C). Furthermore, at any given time, violence is also irregularly distributed throughout the country: some places have no records of drug-war-related crime, while in other places the death toll is higher than in many war-ridden countries in the world [18]: for example, the city of Juárez, in the state of Chihuahua, had more than 300 casualties in one month, a number superior to the average number of deaths in countries with civil war like Syria or Iraq [18]. However, there is some kind of statistical order behind this inhomogeneity. Fig 2 shows the rank–frequency of total death rate by city [19], the curve is well fitted by a power law, with an exponent *β* ≈ −1.57. This result is reminiscent of those found already by L.F. Richardson in his seminal works in relation to the size and frequency of conflicts [12, 13].

### Geographical spread

**Fig 3:**
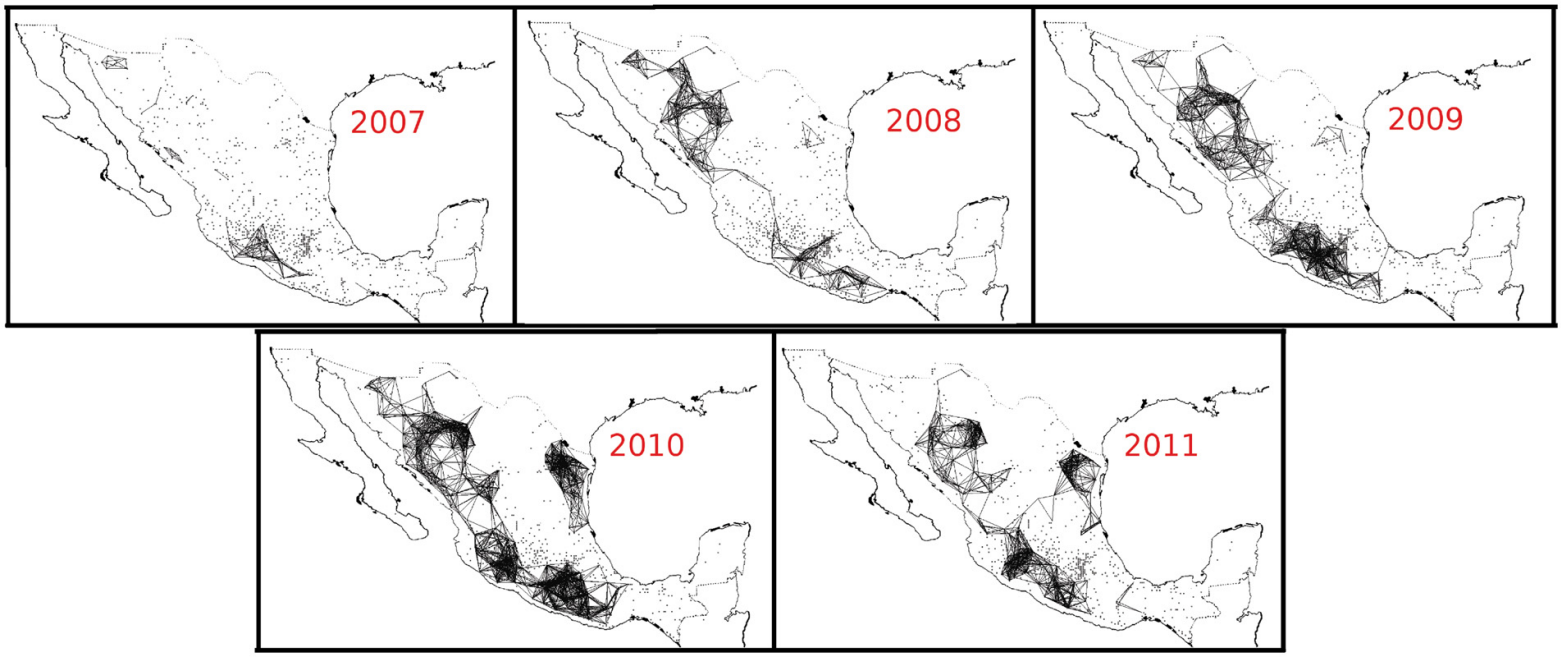
Map of the spread of violence since 2007 to 2011. Each line joins municipalities with more than 70 casualties per 100,000 inhabitants each year and have municipal government seats located closer than 200 km apart. Points are municipalities with at least one narco-related death that year. In 2007 there were only small groups of connected cities in the states of Michoacán, Guerrero, Sinaloa and Chihuahua. During the following months, the Pacific coast suffered a burst of violence, while the Northwestern part (Nuevo León and Tampaulipas) did not present a significant increase of violent episodes. However, at the end of 2009 and beginning of 2010, that part of the country was whipped with internal struggle supposedly between the Gulf Cartel and its former gunned arm, the Zetas group. By the period 2010–2011, it is possible to cross the country visiting only neighboring violent municipalities.

One of the most terrible effects of the entrance of the Mexican army into this conflict was the spread of violence around the alleged principal drug-trade centers: Michoacán, Sinaloa and Chihuahua. Fig 3 shows how violence has spread across the country: in each panel a straight line is drawn between municipalities which have a death rate higher than 70 casualties per 100,000 inhabitants each year (approximately the same rate as in Iraq during the US invasion [18]) and the seats of their municipal governments are less than 200 km apart (a rather arbitrary, but reasonable, distance to travel quickly from one town to the other). In 2007, the links were mostly limited to the states of Michoacán, Guerrero, Sinaloa and Sonora. During 2008, the violence percolated along the Pacific coast (the principal places of operations of the Sinaloa Cartel and the Familia Michoacana Cartel). By 2010, death rates higher than 70 per 100,000 inhabitants had reached the northeastern part of México: Tamaulipas and Nuevo León. By 2011, it was possible to travel from East to West and from North to South across the country by visiting only cities with death rates > 70 and closer than 200 km.

## Results

### The Narco-war Network

The structure and localization of conflicts between organized crime groups is often difficult to identify, since they usually do not advertise their identities and it is not possible to have direct access to the group’s plans and actions. In some cases, the states in which specific drug cartels operate at certain moments are known [20, 21]; however, it is not known how the groups expand or change place of action. We propose that the localization of determined groups, or more specifically conflicts between groups, may be inferred by analyzing the *changes* in the number of drug-related casualties in the various municipalities as a function of time. In order to identify the underlying structure of this war, we construct a correlation network by using the monthly change in the number of death people in each municipality along the years for which we have data.

We calculate the joint variation in the change in number of organized-crime-related deaths in each month with respect to the national average that month, between every pair of municipalities in the country and normalized by the country-wide variance that month times the number of municipalities in the country. Specifically, between each pair of municipalities we calculate

$$\begin{array}{c} SDCC_{\left(X_{i},Y_{i}\right)}=\frac{\left[\left(X_{i}\right)\left(Y_{i}\right)-\left({\mu_{i}}^{2}\right)\right]}{N\sigma_{i}}, \end{array}$$

where *X*_*i*_ and *Y*_*i*_ denote the change in the number of casualties at each municipality during the month *i* with respect to the previous month; *μ*_*i*_ and *σ*_*i*_ denote the average and variance over all cities during the *i* ^*th*^ month, respectively, and *N* is the number of municipalities in the country. This number represents a “single datum correlation coefficient” (SDCC). Next, connecting the cities for which this SDCC is above a certain threshold *θ* allowed us to generate a monthly network of “correlated” municipalities from January 2007 to September 2011 (for more information about the analysis, see the Materials section). This network simply connects the sites in which there is a simultaneous strong increase (or decrease) of organized crime-related violence.

**Fig 4:**
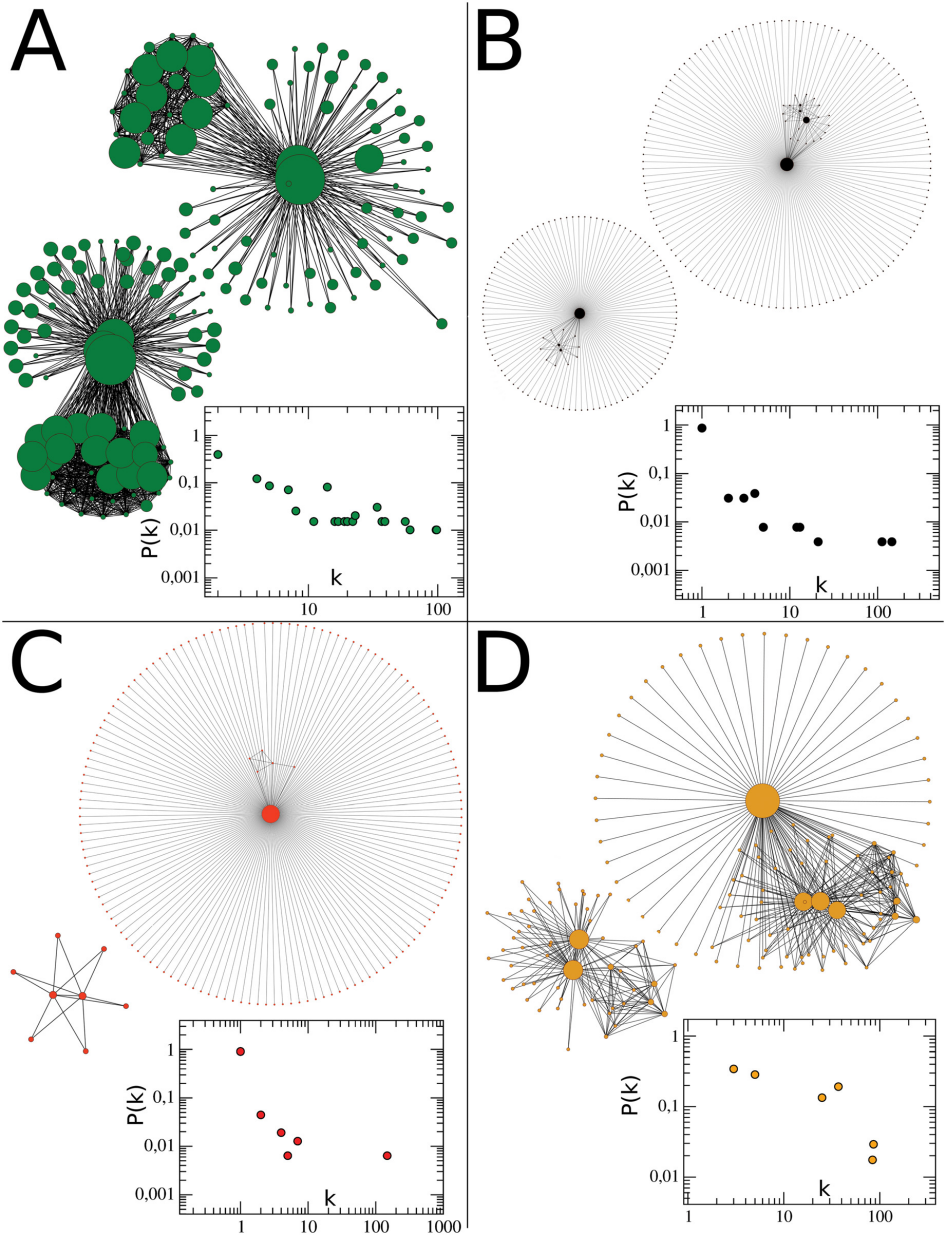
Typical topologies of networks obtained by the correlations between municipalities with organized-crime-related casualties. A) Network obtained by correlations in November 2007. B) August 2008; C) December 2008; D) July 2010. In each network the size of nodes represent the degree of each node. Colors serve as identifiers of each type of network in the rank order plots of Figs 5 and 6 [24].

**Fig 5:**
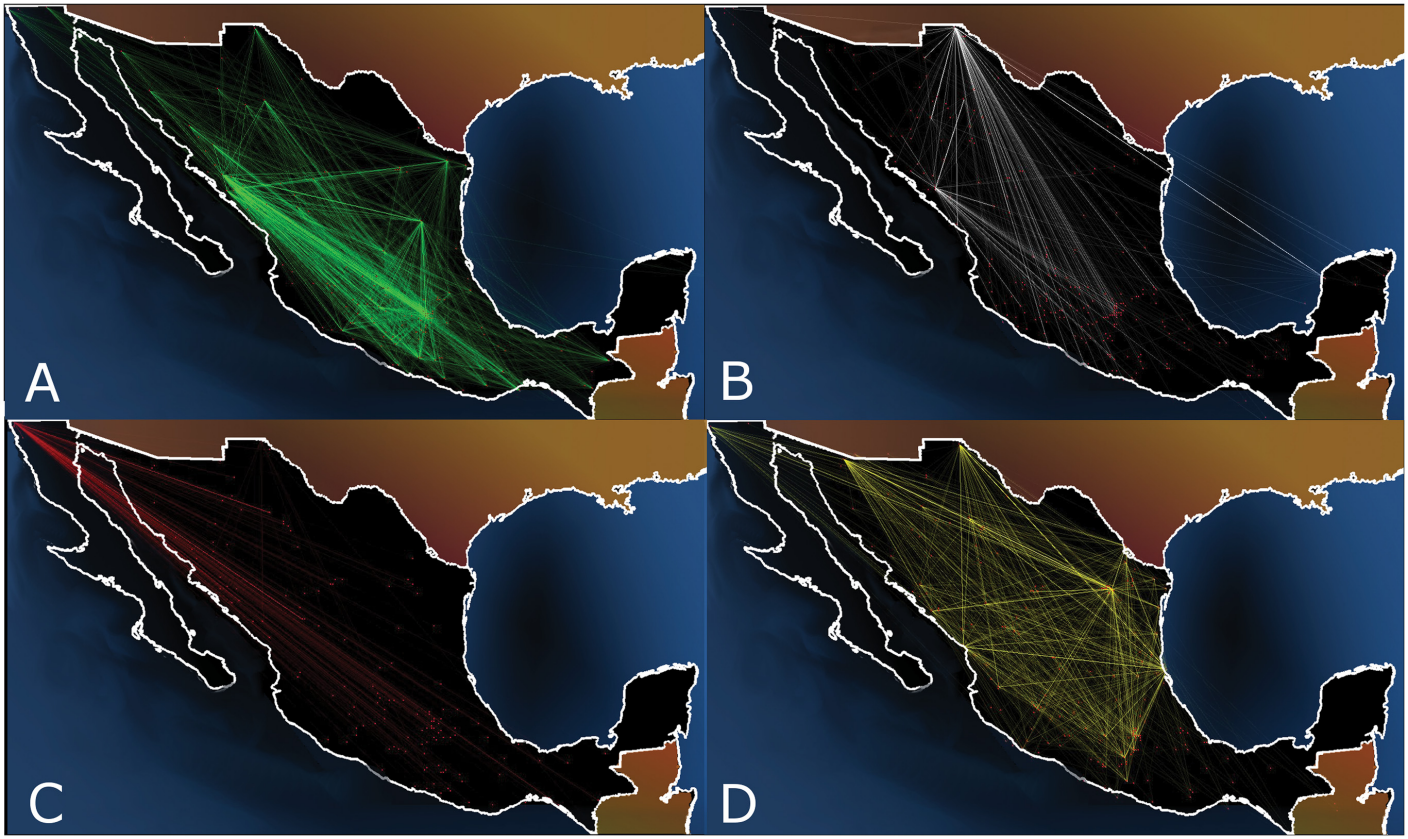
Map representing correlations corresponding to typical topologies of networks during certain months. Red points are municipalities which present at least one casualty due to the organized crime during that month. Two points are linked by a colored line if the correlation between them is bigger than a fixed threshold value. Figures A) to D) represent November 2007; August 2008; December 2008 and July 2010, respectively. The color code corresponds to that used in Fig 4. Brightness of colors are according to the correlation value. It is clear the differences between months, reflecting the non-stationarity of the conflict.

**Fig 6:**
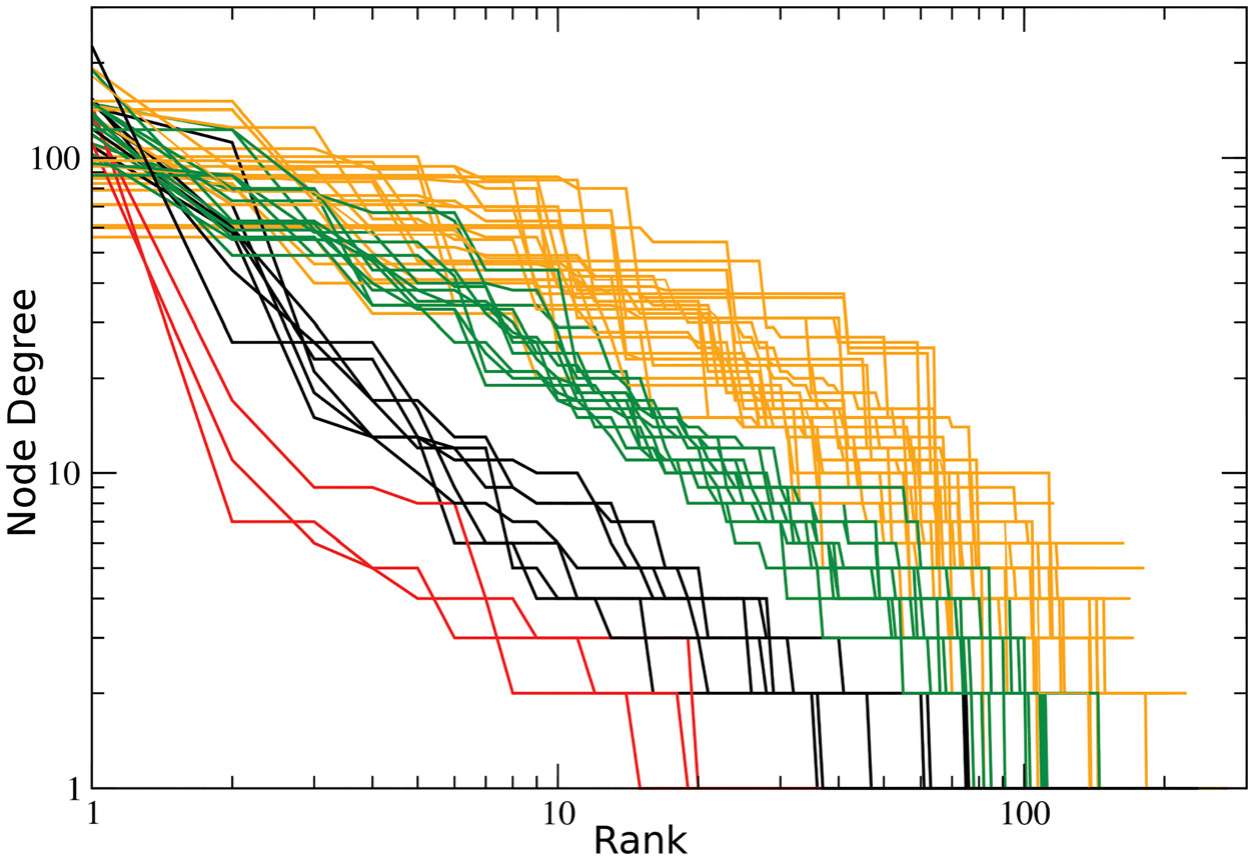
Log–log plot of the degree rank order of the network nodes for each month. Each curve represents the degree of each network node for every month, sorted by its rank. Notice that different types of network topology identified in Fig 4 group together in this plot (colors correspond to those in Fig 4).

**Fig 7:**
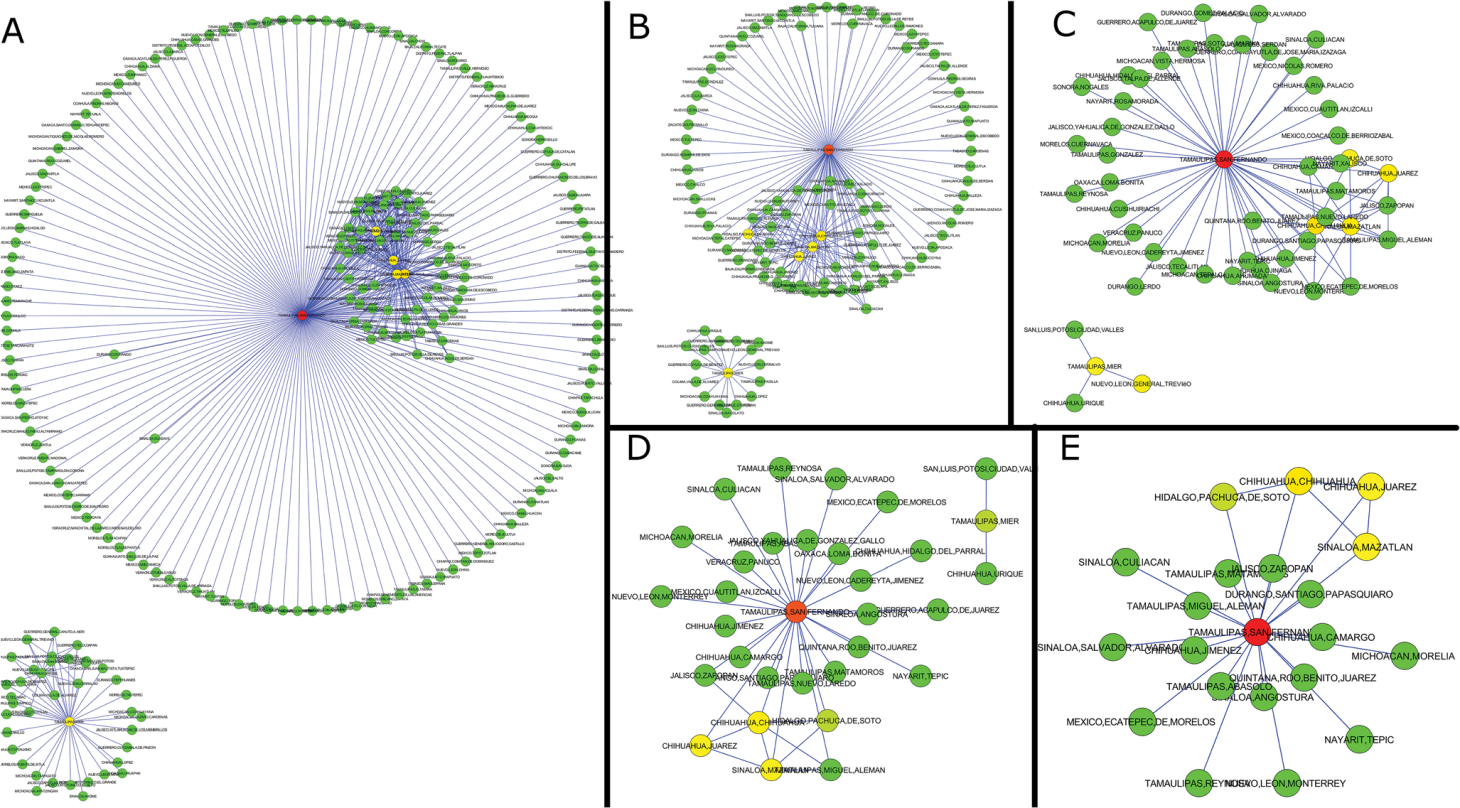
Network topology depending on the threshold value. The networks are depicted at different values of threshold *θ*. A) 0.005; B) 0.01; C) 0.02; D) 0.03 E) 0.04. The color of the nodes (green-yellow-red) depends on their betweenness centrality. It can be observed that the topology is qualitatively conserved: The networks contain a large component in which we have a central node connected to a group of interconnected nodes as well as to a relatively large number of single nodes. Except for panel E, the network contains a second smaller starlike component. This component is not present in panel E due to the fact that the correlation values of the small component are lower than 0.04.

The changes in the intensity and location of the conflicts produce changes in correlation network topologies. In Fig 4, we show the principal topologies that the networks acquired. Figs. A–D are the networks of November 2007, August 2008, December 2008 and July 2010, respectively (with the respective degree distribution in the insets [22, 23]). In Fig 5 we depict the correlation networks corresponding to those shown in Fig 4 as they appear on a map of the country. The first type of network, marked in green, consists of two separated components, each containing several highly-connected centres. Next, we have the network in which nodes are marked in black, which again has two separated components consisting of centres, both of which are highly connected to other less-connected nodes. In the red case, we have a “central” node that is highly connected to many other nodes, and a very small second component of correlated municipalities separated from the large component. Finally, the network marked in yellow consists of a component which is still relatively highly connected to other less-connected municipalities and a separate component which has several multiply-connected nodes. The different types of networks can be observed to form groups, marked with the corresponding colors, in the degree rank order plot (Fig 6) of the networks obtained from the correlations in each month. It is worth pointing out that the network topologies are essentially conserved over a wide range of values of the threshold *θ*. As an example, Fig 7 contains the correlation networks during September of 2010 for different values of *θ* (0.005, 0.01, 0.02, 0.03 and 0.04). As can be observed, the network topology is qualitatively the same up to a correlation threshold value of 0.04 at which point the second component disappears. All correlations between municipalities along the whole period under study at different values of *θ* is provided as a supplementary file (S1 Compressed file).

**Fig 8:**
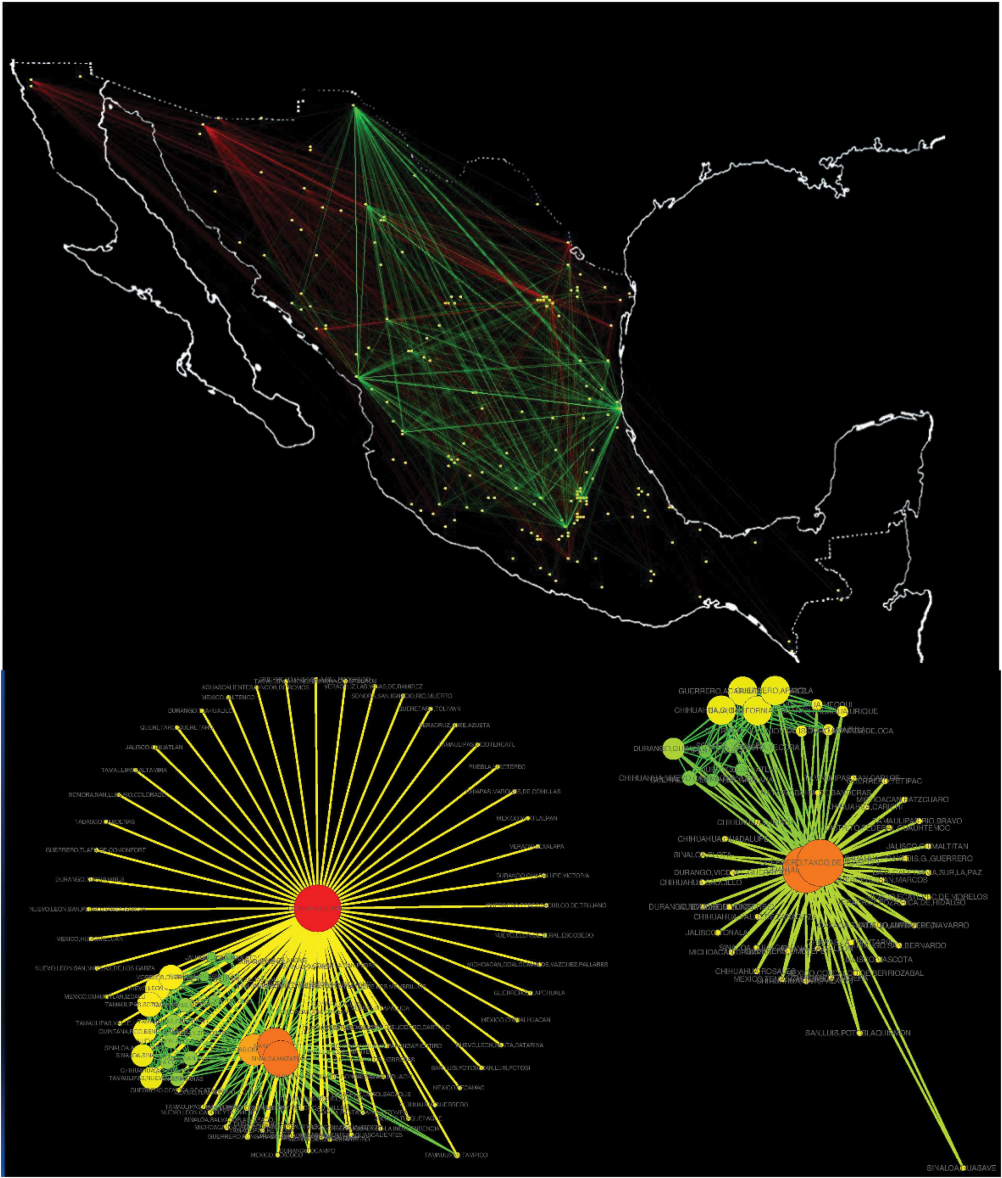
Geographical localization of organized-crime-affected municipalities and correlations amongst them in México. A) Map of México representing the cities affected by organized crime in May of 2010. Yellow points are the municipalities in the network, each component is identified by color: Component-1 links are depicted in green. Component 2 in red. B) Network topology of the correlation network in may of 2010. In this graph, colors indicate betweenness centrality: Red is high betweenness while green nodes represent those with a low betweenness; the size of each node correspond to its degree.

Despite the different topologies, for the threshold values we used in this work, networks always have two separated components; this is due to the fact that we only connect positively correlated nodes. Thus, municipalities belonging to each component are correlated amongst each other and negatively correlated to those in the other component. It is worth pointing out, however, that the two unconnected components into which the narco-network separates are not actually separated geographically, but rather are intertwined (Fig 8). The color of the nodes in the networks in Fig 8 represent the betweenness centrality of each node [24, 25]. This quantity is the number of (shortest) paths connecting nodes in the network that pass through each given node. In communication networks, removing the nodes with highest betweenness centrality would be the most efficient way to disrupt communications. In the context of these narco-war networks, high betweenness centrality might single out the main stages in which the conflict was taking place at each time. This identification may be trivial in the cases of network components that are essentially star-like [25], but it may be much more delicate in components like those shown in the figure.

The city of Juárez is the only municipality appearing in the network every month; we denote the component that contains Juárez as component 1, and the component not containing Juárez as component 2. The rest of the nodes may appear in one component some months, then shift to the other. These shifts, together with the geographical interwining of the network components, suggest that the components, or more precisely, the conflicts that these components represent, are not strongly linked to the spatial distribution of cities in México.

### Prevalence of a small main core of municipalities in each component and important events

As mentioned above, the data analyzed here does not appear to be stationary, thus averaging over several months cannot be justified. However, it is probably the case that many of the correlations detected in each month are fortuitous and should be removed. By analyzing the nodes of each component in 6-month windows, we found the presence of a small group of municipalities in each component of the network that appear consistently during each period: we call these the network *core*. Specifically, municipalities are marked as belonging to the core if they appear in the same network component at least four out of the six months, in each 6-month period; those marked in red belong to component 1, which, as mentioned above, contains Juárez, the only municipality to appear throughout the whole period under consideration; those belonging to component 2 are marked in black.

**Fig 9:**
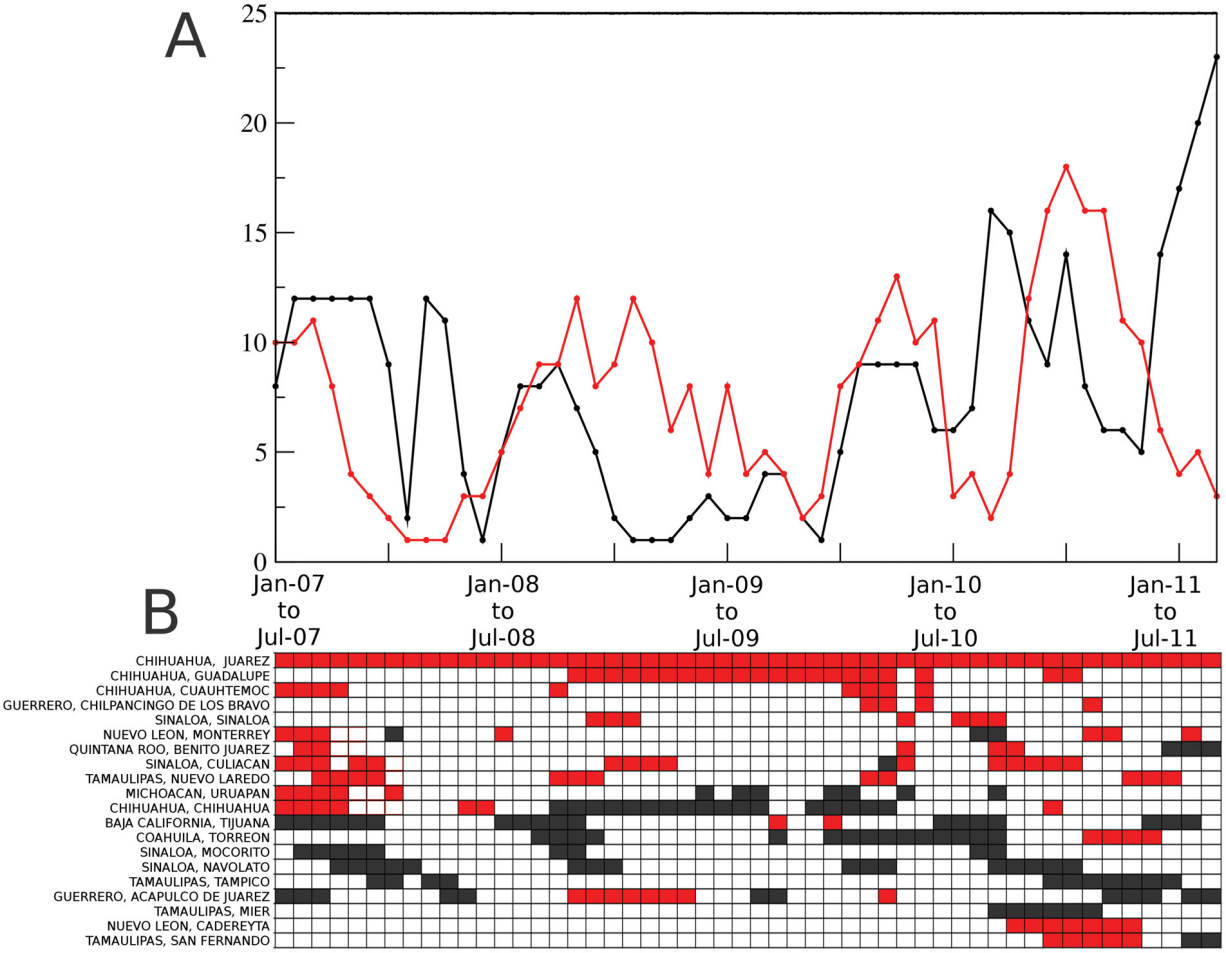
Persistence of municipalities in a network component for 4 out of 6 months: network cores. A) Points denote the number of core nodes. The red line corresponds to the component which contains the city of Juárez, Chihuahua, the other component is drawn in black. B) Is a partial list of some of the cities which appear frequently in each core. The cities are ordered so that “groups” in B) correspond with some of the salient features in A). Each black and red square represents a municipality which appears in the same component in at least four out of six networks corresponding to the following six months.

In Fig 9A we show the total number of nodes in each core. Some of the municipalities appearing in these cores are listed in 9b; we have arranged them to highlight the behavior of various groups of municipalities: The first few municipalities appear almost exclusively in core 1; the next two, Uruapan and Chihuahua, move from core 1 to core 2, whereas from Tijuana to Tampico they belong almost exclusively to core 2. At the end of the list we include municipalities from the Northeast of the country, that enter the conflict after July 2010, as violence spread to that region. Acapulco is an example of a city that shifts, for relatively large periods of time, from one core to the other, and back.

### Core cities and related violence

As can be observed in Fig 9, significant increases of highly-correlated core municipalities appear at various stages of the period under study. The first high value occurs at the beginning of 2007. In this case, the core number 1, which contains Juárez, Chihuahua (red), presents a majority of municipalities that belong to northern México (the exception being the city of Uruapan, where the first operation against the drug cartels, **Operación Michoacán**, was launched [1]). Since each black or red square from Fig 9B represent 6-month periods beginning on that date, one can conclude that the first period of high violence lasted around one year). The second increase in violence in core 1 occurs around the second half of 2008. While it would be rather speculative to assign causes to the different periods of extended conflict, we can mention some of the publicly known highlights. For example, during this second period there were two important events related to the war-against drug: the apprehension of Alfredo Beltrán Leyva, leader of the Sinaloa cartel. The other relevant event was the granade attack in Morelia, Michoacán, during the celebration of the Mexican Independence Day. In December of 2009, Arturo Beltrán Leyva, brother of Alfredo and leader of the Beltrán Leyva cartel, was killed by the Mexican Navy in the City of Cuernavaca, Morelos. This event alledgedly broke the unstable equilibrium prevailing between the drug cartels and might be related with the third rise of violence. Finally, the fourth event, was the schism between the **“Zetas”** group with the **“Cartel del Golfo”** cartel which occurred after the murder of Ezequiel Cárdenas Guillén. Both factions fought each other over the control of the north-eastern border (the states of Nuevo León and Tamaulipas), converting that region of the country into a battle field. This can be observed as a steep increase at the end of Fig 9A and on the bottom right of the list in 9B, where new municipalities in the states of Nuevo León and Tamaulipas appear amongst the core 1 cities. This part of the conflict is also observed in Fig 2, where in the last panels, we can see the spread of violence reach the Northeast region of the country and the Gulf of México. Thus, it appears that the single time correlation used to generate our networks, and the identification of network cores, might be useful tools that highlight temporal relationships between cities affected by the narco-war, despite the nonstationarity of the data. This analysis may help to identify the moments and places in which drug-related conflicts took place, and may suggest which factions were involved. This kind of analysis may also help to describe the emergence and propagation of gang-related violence waves [26].

## Discussion

In this work we address one of the most dreadful situations in México during the last few years: The increase and spread of violence related with organized crime thoughout the country. Using the official monthly number of drug-cartel-related casualties in each municipality of México during the period comprised between January of 2007 and September of 2011, we analyze the spread and evolution of the conflicts between the mexican drug cartels amongst each other, as well as with the mexican armed forces. Since the data is non-stationary, we construct networks based on the correlation in the *changes* of the monthly number of casualties attributed to drug cartels, between different municipalities. We find that close geographical distance between violent cities does not imply a strong correlation amongst them. Additionally, we observe that conflict evolves in relatively short-term periods, in which a small core of violent cities determines the main theatre of the war at each stage; the networks are consistent with the direct observation on how violence spread throughout the country. The geographical localization of these *core cities*, seems to indicate that each network component is related with conflicts involving particular criminal groups.

The problem of organized crime in general, and narcotraffic in particular, together with the associated violence, homicides, kidnapping, extortion, etc., are very complex phenomena, which have proven to be extremely difficult to analyze beyond the accumulation of simple, though alarming, statistics. The construction and use of tools to extract information from the available data may aid in the efforts to unveil the behavior and evolution of these patterns of violence, with the (still distant) hope to design strategies which reduce this dismal situation.

## Materials

### Data Acquisition

We obtained the number of drug-related deaths by municipality from December 2006 to December 2010 from the Mexican presidency web page. The data from January 2011 to September 2011 was obtained from the web page of the Attorney General’s Office (PGR). As mentioned in the text, these data are no longer available. We provide the complete database in S1 Dataset (comprising Jan. 2007–Dec. 2010) and S1 and S2 Tables (from Jan. 2011–Sep. 2011). We present the data in the same format that they were originally provided. Population data was obtained from the National Institute of Statistics, Geography and Informatics (INEGI) [27]. The geographical coordinates of each municipal government seat were obtained from Google Maps.

### Network Construction

The narco-network nodes are the municipalities affected by the organized crime between 2007 and 2011. The network was constructed by using the correlation in the monthly change in the number of casualties in each place (SDCC), as defined in the Results section. Specifically, two nodes (municipalities) *X* and *Y* are linked if they satisfy

$$\begin{array}{c} SDCC_{\left(X_{i},Y_{i}\right)}>\theta \end{array}$$

where *θ* is th correlation threshold. In the case of Fig 4 the value of *θ* is 0.005. The magnitude of *θ*, merely reflects the way we chose to normalize the SDCC. The actual value was chosen low enough as to never have empty networks. The qualitative features of the networks and the identity of the core cities is not very dependent on the precise value of *θ*. We constructed 57 networks, one for each month. Networks were depicted using CytoScape version 2.7.1 [24].

## Supporting Information

S1 Dataset Table with the number of casualties by month related to organized crime in México from December 2006 to December 2010. This table was obtained from the Mexican presidency web page: http://www.presidencia.gob.mx/base-de-datos-de-fallecimientos/ (no longer available online). The list is ranked from the most violent municipality (Juárez, Chihuahua) to the least violent. Each entry contains the name of the state and the name of municipality, the coordinates of the municipal government seat, its population in 2010, the total number of casualties during those 4 years, the rate per 100,000 inhabitants, and the number of casualties each month.
(XLS)

S1 Table Table with the number of casualties by month related to the organized crime in México from January 2011 to September 2011. Table obtained by the general attorney’s office (PGR) web page: http://www.pgr.gob.mx/temas%20relevantes/estadistica/FALLECIMIENTOS%20POR%20PRESUNTA%20RIVALIDAD%20DELINCUENCIAL%202011%20%28Enero-Septiembre%29.pdf (no longer available online). Each entry contains the name of the state, the municipality and the number of casualties per month. The totals for the 9 months are given at the end of the file
(PDF)

S2 Table Table with the number of casualties by month related to the organized crime in México from January 2011 to September 2011. We also provide this table which contains the same information than S2 Table but in a friendlier spreadsheet format.
(XLS)

S1 Compressed file Correlation values by month between municipalities depending on the value of *θ*. Each file contains all the correlations above certain threshold (indicated in the name of the file), during a particular month (also indicated in the name of the file). The file names have the value of *θ* followed by the number of the *i* ^*th*^ month, starting from January of 2007 until Sept. 2011.
(ZIP)
