# Supplementary material for: Analysis of México’s Narco-War Network (2007–2011)
Source: PLoS One. 2015 May 18;10(5):e0126503. doi: 10.1371/journal.pone.0126503 (PMC4436224; doi:10.1371/journal.pone.0126503)
Supplement: S1 Table — Table obtained by the general attorney’s office (PGR) web page: http://www.pgr.gob.mx/temas%20relevantes/estadistica/FALLECIMIENTOS%20POR%20PRESUNTA%20RIVALIDAD%20DELINCUENCIAL%202011%20%28Enero-Septiembre%29.pdf (no longer available online). Each entry contains the name of the state, the municipality and the number of casualties per month. The totals for the 9 months are given at the end of the file (PDF) [file pone.0126503.s002.pdf]

TOTAL FALLECIMIENTOS POR PRESUNTA RIVALIDAD DELINCUENCIAL
ENERO - SEPTIEMBRE 2011

ENTIDAD FEDERATIVA	MUNICIPIO	ENERO	FEBRERO	MARZO	ABRIL	MAYO	JUNIO	JULIO	AGOSTO	SEPTIEMBRE	TOTAL
AGUASCALIENTES	AGUASCALIENTES	6	6	3	4	3	4	2	1		29
AGUASCALIENTES	EL LLANO		1		1						2
AGUASCALIENTES	JESUS MARIA		1	1		2	1				5
AGUASCALIENTES	PABELLON DE ARTEAGA	1		1			2			1	5
BAJA CALIFORNIA	ENSENADA		2			1	2	2		2	9
BAJA CALIFORNIA	MEXICALI	7	2	7	2	1	3	1	2	4	29
BAJA CALIFORNIA	PLAYAS DE ROSARITO	2		4	3	3	1	2		4	19
BAJA CALIFORNIA	TECATE	4		1	1	1		2		1	10
BAJA CALIFORNIA	TIJUANA	23	12	29	22	24	20	14	20	19	183
BAJA CALIFORNIA SUR	COMONDU	1									1
BAJA CALIFORNIA SUR	LA PAZ						2	1	1		4
BAJA CALIFORNIA SUR	LORETO	1									1
BAJA CALIFORNIA SUR	LOS CABOS				1				3		4
CAMPECHE	CALAKMUL	1									1
CAMPECHE	CALKINI								1		1
CAMPECHE	CARMEN		1	4	2	1					8
CHIAPAS	ACAPETAHUA			1							1
CHIAPAS	BENEMERITO DE LAS AMERICAS						2		1		3
CHIAPAS	CATAZAJA			1							1
CHIAPAS	CHAMULA							1			1
CHIAPAS	CHIAPA DE CORZO						1				1
CHIAPAS	COMITAN DE DOMINGUEZ	2									2
CHIAPAS	FRONTERA HIDALGO		1								1
CHIAPAS	HUEHUETAN	3									3
CHIAPAS	HUITIUPAN	1									1
CHIAPAS	HUIXTLA	1	1	2	1			1			6
CHIAPAS	IXTACOMITAN		1								1
CHIAPAS	IXTAPA								2		2
CHIAPAS	JIQUIPILAS			1							1
CHIAPAS	LA TRINITARIA	1									1
CHIAPAS	LAS MARGARITAS	1									1
CHIAPAS	MARQUES DE COMILLAS					1					1
CHIAPAS	OCOSINGO						1				1
CHIAPAS	OCOZOCOAUTLA DE ESPINOSA			1							1
CHIAPAS	PALENQUE					1					1
CHIAPAS	PANTELHO				1						1
CHIAPAS	PUEBLO NUEVO SOLISTAHUACAN	2	1					1			4
CHIAPAS	REFORMA				4			1			5
CHIAPAS	SAN CRISTOBAL DE LAS CASAS								1		1
CHIAPAS	SUCHIATE				3	1		1	2		7
CHIAPAS	TAPACHULA		3		3	1		1	2	1	11
CHIAPAS	TUXTLA CHICO			1							1
CHIAPAS	VENUSTIANO CARRANZA				1						1
CHIAPAS	VILLAFLORES								1		1
CHIHUAHUA	AHUMADA	2		1		2	1				6
CHIHUAHUA	ALDAMA				2		1	1	1		5
CHIHUAHUA	ALLENDE				1						1

CHIHUAHUA	AQUILES SERDAN			2					2	2	6
CHIHUAHUA	ASCENSION	5	3	2	4	1			2	2	19
CHIHUAHUA	BALLEZA	1	1		2	6	1		1		12
CHIHUAHUA	BATOPILAS						2				2
CHIHUAHUA	BOCOYNA	1		1		2	1	7	3		15
CHIHUAHUA	BUENAVENTURA									1	1
CHIHUAHUA	CAMARGO	7	3		5	7	5	1	6	4	38
CHIHUAHUA	CARICHI		1						1	1	3
CHIHUAHUA	CASAS GRANDES	1	1	3		3				1	9
CHIHUAHUA	CHIHUAHUA	28	29	56	62	49	37	51	58	32	402
CHIHUAHUA	CHINIPAS				2		2				4
CHIHUAHUA	CUAUHTEMOC	4		2	2	1	5	3		4	21
CHIHUAHUA	CUSIHUIRIACHI	1	2	4	1		2	4		1	15
CHIHUAHUA	DELICIAS	7	5	9	2	4	4	6	8	14	59
CHIHUAHUA	DOCTOR BELISARIO DOMINGUEZ								1		1
CHIHUAHUA	GOMEZ FARIAS					1					1
CHIHUAHUA	GUACHOCHI	3	1	2	3	2	5	2	10		28
CHIHUAHUA	GUADALUPE	6		5		2	4	4	1	2	24
CHIHUAHUA	GUADALUPE Y CALVO	9			2	3	5	5	7	1	32
CHIHUAHUA	GUAZAPARES					2				5	7
CHIHUAHUA	GUERRERO	1		2			1		1	1	6
CHIHUAHUA	HIDALGO DEL PARRAL	13	10	10	6	4	10	5	7	12	77
CHIHUAHUA	JANOS	2					1				3
CHIHUAHUA	JIMENEZ	11	11	5	5	4	4	2	2	3	47
CHIHUAHUA	JUAREZ	165	192	126	125	96	120	166	96	120	1,206
CHIHUAHUA	JULIMES						3				3
CHIHUAHUA	LA CRUZ	3	1		2						6
CHIHUAHUA	LOPEZ	1									1
CHIHUAHUA	MADERA	3				1	1		1	13	19
CHIHUAHUA	MATAMOROS	1		3					2		6
CHIHUAHUA	MEOQUI	6	1	1	6	1	3	6	10	9	43
CHIHUAHUA	MORELOS	1	2			1		1			5
CHIHUAHUA	MORIS						8		1		9
CHIHUAHUA	NAMIQUIPA	1			1						2
CHIHUAHUA	NUEVO CASAS GRANDES	1	1	2	2		1		1		8
CHIHUAHUA	OCAMPO			1	1	2		2	2	2	10
CHIHUAHUA	OJINAGA	1		2	1		1	1			6
CHIHUAHUA	PRAXEDIS G. GUERRERO			2		2	1				5
CHIHUAHUA	RIVA PALACIO	1				2					3
CHIHUAHUA	ROSALES	2	3	3	4	2	3	4	2	8	31
CHIHUAHUA	SAN FRANCISCO DE BORJA			1							1
CHIHUAHUA	SAN FRANCISCO DE CONCHOS	2		1							3
CHIHUAHUA	SAN FRANCISCO DEL ORO									4	4
CHIHUAHUA	SANTA ISABEL				1						1
CHIHUAHUA	SATEVO				4						4
CHIHUAHUA	SAUCILLO	4		1	2	1	7	2	2		19
CHIHUAHUA	TEMOSACHIC				1	2	7			1	11
CHIHUAHUA	URIQUE	5	1	4	2	4			1	2	19
CHIHUAHUA	URUACHI			3		4	2		2	1	12
CHIHUAHUA	VALLE DE ZARAGOZA	2				2	4				8
COAHUILA	ACUÑA				1					1	2
COAHUILA	FRANCISCO I. MADERO			1		1		1	2		5
COAHUILA	FRONTERA						2				2

COAHUILA	GENERAL CEPEDA						1				1
COAHUILA	MATAMOROS							1			1
COAHUILA	MONCLOVA					3	5	4			12
COAHUILA	PARRAS					1					1
COAHUILA	PIEDRAS NEGRAS							1		4	5
COAHUILA	RAMOS ARIZPE	1		4	3			1			9
COAHUILA	SALTILLO	3	2	13	1	8	5	4	9	5	50
COAHUILA	SAN JUAN DE SABINAS						1				1
COAHUILA	SAN PEDRO			1							1
COAHUILA	TORREON	36	44	26	34	49	80	67	53	87	476
COAHUILA	VIESCA			5	1						6
COLIMA	ARMERIA	1	4			1		1	2	2	11
COLIMA	COLIMA	2	5	5	1	4	9	1	4	4	35
COLIMA	COMALA		2	1		2					5
COLIMA	COQUIMATLAN					2		1			3
COLIMA	IXTLAHUACAN					1					1
COLIMA	MANZANILLO	1		2		3		1	2	1	10
COLIMA	TECOMAN	2	2	1	1	1	2	3	2	1	15
COLIMA	VILLA DE ALVAREZ	1	2	2		1	1	3	3	1	14
DISTRITO FEDERAL	ALVARO OBREGON	3							1		4
DISTRITO FEDERAL	AZCAPOTZALCO	1				1					2
DISTRITO FEDERAL	BENITO JUAREZ				1					1	2
DISTRITO FEDERAL	COYOACAN	1	2		1				1		5
DISTRITO FEDERAL	CUAUHTEMOC	4		4	3	1	2	2	2		18
DISTRITO FEDERAL	GUSTAVO A. MADERO	1	2	2	1	5	3	1	2	2	19
DISTRITO FEDERAL	IZTACALCO		2			1		1	1		5
DISTRITO FEDERAL	IZTAPALAPA	9	3	1	5	2	1	4	5	1	31
DISTRITO FEDERAL	LA MAGDALENA CONTRERAS				1						1
DISTRITO FEDERAL	MIGUEL HIDALGO			1	1			1	1		4
DISTRITO FEDERAL	TLAHUAC		2		1				1		4
DISTRITO FEDERAL	TLALPAN		3	2			2	1	1		9
DISTRITO FEDERAL	VENUSTIANO CARRANZA	3	1	2	2	2			2	3	15
DISTRITO FEDERAL	XOCHIMILCO	1			1			1			3
DURANGO	CANATLAN	3	1				8				12
DURANGO	CONETO DE COMONFORT			1		5					6
DURANGO	CUENCAME	5	2						11	3	21
DURANGO	DURANGO	2	4	24	123	187	34	5	4	7	390
DURANGO	EL ORO	1	2								3
DURANGO	GOMEZ PALACIO	32	3	10	16	13	7	14	10	11	116
DURANGO	GUADALUPE VICTORIA							1			1
DURANGO	GUANACEVI			1	1				1		3
DURANGO	LERDO	10	4	4	4		3	2	10	3	40
DURANGO	MAPIMI			2	3						5
DURANGO	MEZQUITAL	1	1		3	2					7
DURANGO	NOMBRE DE DIOS				2	1					3
DURANGO	NUEVO IDEAL		1				2				3
DURANGO	OCAMPO			3	2						5
DURANGO	OTAEZ						2				2
DURANGO	POANAS	8		2				3			13
DURANGO	PUEBLO NUEVO	1			2		1			2	6
DURANGO	RODEO							6	2		8
DURANGO	SAN DIMAS				4	8	1	6			19
DURANGO	SAN JUAN DEL RIO	1									1

DURANGO	SANTIAGO PAPASQUIARO	1		2	12	2	1	4	1		23
DURANGO	TAMAZULA		2					1		3	6
DURANGO	TEPEHUANES	4		1	1					1	7
DURANGO	TLAHUALILO			1		1					2
DURANGO	TOPIA									2	2
DURANGO	VICENTE GUERRERO				1	3		1			5
GUANAJUATO	ABASOLO									1	1
GUANAJUATO	ACAMBARO	2	2		2		2	2			10
GUANAJUATO	APASEO EL ALTO		2		1		5				8
GUANAJUATO	APASEO EL GRANDE						1	1		2	4
GUANAJUATO	CELAYA	3	3	2	1	8	6	1	3	6	33
GUANAJUATO	CORTAZAR		2	1							3
GUANAJUATO	CUERAMARO					1					1
GUANAJUATO	IRAPUATO	1		1	3					1	6
GUANAJUATO	JARAL DEL PROGRESO			1				2	1		4
GUANAJUATO	JERECUARO	1		2	2						5
GUANAJUATO	LEON	1	3	1	2		7	3	1	2	20
GUANAJUATO	MOROLEON	1								1	2
GUANAJUATO	OCAMPO					2					2
GUANAJUATO	PENJAMO	1	1		1				1		4
GUANAJUATO	ROMITA									1	1
GUANAJUATO	SALAMANCA		1	2	3	2		1	4	2	15
GUANAJUATO	SALVATIERRA				1						1
GUANAJUATO	SAN DIEGO DE LA UNION	2									2
GUANAJUATO	SAN FELIPE		1								1
GUANAJUATO	SAN JOSE ITURBIDE	2									2
GUANAJUATO	SAN LUIS DE LA PAZ	1	1								2
GUANAJUATO	SANTA CRUZ DE JUVENTINO ROSAS				3				1	1	5
GUANAJUATO	SILAO	3	1			1	1	1			7
GUANAJUATO	TARANDACUAO		1		1						2
GUANAJUATO	URIANGATO			1		1		1	1		4
GUANAJUATO	VALLE DE SANTIAGO		1		1		2				4
GUANAJUATO	VILLAGRAN	2									2
GUANAJUATO	YURIRIA			1				1	2		4
GUERRERO	ACAPULCO DE JUAREZ	68	81	91	77	80	71	94	152	81	795
GUERRERO	AJUCHITLAN DEL PROGRESO	2	1		2	3	5	8			21
GUERRERO	ALPOYECA				1	1					2
GUERRERO	APAXTLA	3				3					6
GUERRERO	ARCELIA		1	4	1						6
GUERRERO	ATLAMAJALCINGO DEL MONTE							1			1
GUERRERO	ATLIXTAC					1					1
GUERRERO	ATOYAC DE ALVAREZ	2		5	1	5	11	2	12	3	41
GUERRERO	AYUTLA DE LOS LIBRES	3	1			4	2	1		1	12
GUERRERO	AZOYU							1			1
GUERRERO	BENITO JUAREZ	1		1			1	2			5
GUERRERO	BUENAVISTA DE CUELLAR			7							7
GUERRERO	CHILAPA DE ALVAREZ			2		1	2	3	2		10
GUERRERO	CHILPANCINGO DE LOS BRAVO	3	3	2	3	7	6	7	8	4	43
GUERRERO	COAHUAYUTLA DE JOSE MARIA IZAZAGA	2			1			2			5
GUERRERO	COCHOAPA EL GRANDE		3					1			4
GUERRERO	COCULA						2			2	4
GUERRERO	COPALA		1	2							3
GUERRERO	COPALILLO				1						1

GUERRERO	COPANATOYAC				5						5
GUERRERO	COYUCA DE BENITEZ	4	3	3	1	4	6	2	2	1	26
GUERRERO	COYUCA DE CATALAN	18	2	3	5	4	7	9	1	3	52
GUERRERO	CUAJINICUILAPA	1		1	3	4				2	11
GUERRERO	CUAUTEPEC									1	1
GUERRERO	CUTZAMALA DE PINZON	1	1			1	2		6	1	12
GUERRERO	EDUARDO NERI		5	2	3	4	3	2	3		22
GUERRERO	FLORENCIO VILLARREAL					1		2			3
GUERRERO	GENERAL CANUTO A. NERI					1					1
GUERRERO	GENERAL HELIODORO CASTILLO			1	2		6	4		6	19
GUERRERO	HUITZUCO DE LOS FIGUEROA	1						2		6	9
GUERRERO	IGUALA DE LA INDEPENDENCIA	4	5	3	1	6		16	2		37
GUERRERO	JUAN R. ESCUDERO				2			1			3
GUERRERO	LA UNION DE ISIDORO MONTES DE OCA	1	1				1				3
GUERRERO	LEONARDO BRAVO					1	1		1		3
GUERRERO	MARQUELIA	1						1			2
GUERRERO	MARTIR DE CUILAPAN	2	4			1				1	8
GUERRERO	MOCHITLAN							1			1
GUERRERO	OMETEPEC	1				1	2	3	3	1	11
GUERRERO	PETATLAN			9	6	1	1	2	2	1	22
GUERRERO	PUNGARABATO	9	4		1	7	1	5		1	28
GUERRERO	QUECHULTENANGO	5	1	1		1	1				9
GUERRERO	SAN LUIS ACATLAN			1							1
GUERRERO	SAN MARCOS	1	1	6	1		2	1		1	13
GUERRERO	SAN MIGUEL TOTOLAPAN	1	5	8			4				18
GUERRERO	TAXCO DE ALARCON		3	1	8	7	1	8			28
GUERRERO	TECOANAPA							1			1
GUERRERO	TECPAN DE GALEANA	4	3		8	12	2	13	13	13	68
GUERRERO	TELOLOAPAN						2	2	1		5
GUERRERO	TEPECOACUILCO DE TRUJANO			7		1	6			3	17
GUERRERO	TETIPAC									1	1
GUERRERO	TIXTLA DE GUERRERO		1					1	1		3
GUERRERO	TLACOACHISTLAHUACA	1		1	1	1	2			1	7
GUERRERO	TLACOAPA						5				5
GUERRERO	TLALCHAPA			1		3					4
GUERRERO	TLAPA DE COMONFORT	1			1	1		1	4	1	9
GUERRERO	TLAPEHUALA					1					1
GUERRERO	XOCHIHUEHUETLAN									2	2
GUERRERO	ZAPOTITLAN TABLAS				1	1		1		1	4
GUERRERO	ZIHUATANEJO DE AZUETA	3	4		16	24	9	10	9	15	90
GUERRERO	ZIRANDARO							3		1	4
GUERRERO	ZITLALA	1									1
HIDALGO	ACATLAN									1	1
HIDALGO	ATOTONILCO DE TULA								1		1
HIDALGO	ATOTONILCO EL GRANDE	2									2
HIDALGO	EPAZOYUCAN		1								1
HIDALGO	HUEJUTLA DE REYES	1		1			1			1	4
HIDALGO	MINERAL DE LA REFORMA			1							1
HIDALGO	OMITLAN DE JUAREZ						1				1
HIDALGO	PACHUCA DE SOTO	1		1						1	3
HIDALGO	SAN BARTOLO TUTOTEPEC									1	1
HIDALGO	SAN FELIPE ORIZATLAN				1						1
HIDALGO	SANTIAGO DE ANAYA			1							1

HIDALGO	SINGUILUCAN		1								1
HIDALGO	TEPEAPULCO			1							1
HIDALGO	TIZAYUCA		1								1
HIDALGO	TLANALAPA			1							1
HIDALGO	TLAXCOAPAN				1						1
HIDALGO	TULA	1									1
HIDALGO	TULA DE ALLENDE			2	5						7
HIDALGO	ZACUALTIPAN DE ANGELES				1						1
HIDALGO	ZEMPOALA	1		2	1						4
JALISCO	AHUALULCO DE MERCADO					1					1
JALISCO	AMACUECA			2		2		1			5
JALISCO	AMATITAN				1				1		2
JALISCO	AMECA				1				1		2
JALISCO	ARANDAS			4		1		8			13
JALISCO	ATOTONILCO EL ALTO			1	2	8			6		17
JALISCO	AUTLAN DE NAVARRO							1	3		4
JALISCO	AYOTLAN				5		1	10	5	5	26
JALISCO	AYUTLA									2	2
JALISCO	BOLAÑOS					1					1
JALISCO	CAÑADAS DE OBREGON					1					1
JALISCO	CASIMIRO CASTILLO	1	1	1	1						4
JALISCO	CHAPALA			1						2	3
JALISCO	CHIMALTITAN				4		2				6
JALISCO	CIHUATLAN	2						1	1		4
JALISCO	COCULA				1					1	2
JALISCO	CUAUTITLAN DE GARCIA BARRAGAN				1						1
JALISCO	DEGOLLADO		1		3		2			2	8
JALISCO	EL ARENAL							5			5
JALISCO	EL GRULLO							1			1
JALISCO	EL SALTO	2		4	1	1	1	4		1	14
JALISCO	ENCARNACION DE DIAZ						1				1
JALISCO	GOMEZ FARIAS									1	1
JALISCO	GUACHINANGO					1					1
JALISCO	GUADALAJARA	12	12	14	6	6	9	6	10	7	82
JALISCO	HOSTOTIPAQUILLO							1			1
JALISCO	HUEJUCAR							2			2
JALISCO	IXTLAHUACAN DE LOS MEMBRILLOS	3					2				5
JALISCO	IXTLAHUACAN DEL RIO		1		5				1		7
JALISCO	JAMAY							1			1
JALISCO	JESUS MARIA							3	1		4
JALISCO	JILOTLAN DE LOS DOLORES					15	1				16
JALISCO	JOCOTEPEC				1			1	2		4
JALISCO	LA BARCA		1	1			1				3
JALISCO	LA HUERTA			1				1			2
JALISCO	LA MANZANILLA DE LA PAZ						1		1		2
JALISCO	LAGOS DE MORENO	3		1			4	4			12
JALISCO	MAGDALENA					1					1
JALISCO	MASCOTA		1								1
JALISCO	MAZAMITLA						2				2
JALISCO	MEZQUITIC				1	4					5
JALISCO	OCOTLAN	1					1	1	1		4
JALISCO	OJUELOS DE JALISCO	2	1								3
JALISCO	PIHUAMO				2		1			2	5

JALISCO	PONCITLAN	1								1	2
JALISCO	PUERTO VALLARTA	1	3	1	2		5	4	4	8	28
JALISCO	QUITUPAN				2						2
JALISCO	SAN CRISTOBAL DE LA BARRANCA			1			6		1	1	9
JALISCO	SAN IGNACIO CERRO GORDO			1							1
JALISCO	SAN MARTIN DE BOLAÑOS		1								1
JALISCO	SAN MIGUEL EL ALTO				3		1				4
JALISCO	SANTA MARIA DEL ORO (JAL)	1					3				4
JALISCO	SAYULA	2		1							3
JALISCO	TALA	1									1
JALISCO	TAMAZULA DE GORDIANO					5	1				6
JALISCO	TAPALPA								2		2
JALISCO	TECALITLAN			2				1			3
JALISCO	TECOLOTLAN		1							1	2
JALISCO	TENAMAXTLAN	1				2					3
JALISCO	TEOCUITATLAN DE CORONA			3							3
JALISCO	TEPATITLAN DE MORELOS	1		2				1	1	2	7
JALISCO	TEQUILA	1					1				2
JALISCO	TIZAPAN EL ALTO			3	2	3	1				9
JALISCO	TLAJOMULCO DE ZUÑIGA	12			1			5	1	5	24
JALISCO	TLAQUEPAQUE	8	2	10	1	2	7	7	4	5	46
JALISCO	TOMATLAN									2	2
JALISCO	TONALA	3	3	4	2		3	4	1	1	21
JALISCO	TOTOTLAN					3	7	3			13
JALISCO	TUXCUECA							3			3
JALISCO	TUXPAN	1		7							8
JALISCO	UNION DE SAN ANTONIO				1					1	2
JALISCO	ZAPOPAN	6	13	10	7	7	16	13	27	10	109
JALISCO	ZAPOTILTIC			1	1			1			3
JALISCO	ZAPOTITLAN DE VADILLO							1	1	1	3
JALISCO	ZAPOTLAN DEL REY							1		3	4
JALISCO	ZAPOTLAN EL GRANDE				2	1		1		1	5
MEXICO	ACOLMAN					1	1			1	3
MEXICO	ACULCO							1			1
MEXICO	ALMOLOYA DE ALQUISIRAS				1						1
MEXICO	ALMOLOYA DE JUAREZ				1				6	1	8
MEXICO	AMECAMECA				1						1
MEXICO	ATIZAPAN DE ZARAGOZA	1	7					3	1		12
MEXICO	ATLAUTLA		9								9
MEXICO	CHALCO	1	3	3			6	6	5		24
MEXICO	CHICOLOAPAN									6	6
MEXICO	CHIMALHUACAN	1					3			1	5
MEXICO	COACALCO DE BERRIOZABAL			1	1	1		1			4
MEXICO	COATEPEC HARINAS				2						2
MEXICO	COCOTITLAN		1			1					2
MEXICO	COYOTEPEC			1		4				1	6
MEXICO	CUAUTITLAN		4								4
MEXICO	CUAUTITLAN IZCALLI	1	1	4	1	5	3	7	3		25
MEXICO	ECATEPEC DE MORELOS	8	6	12	16	14	15	11	13	12	107
MEXICO	EL ORO		1								1
MEXICO	HUEHUETOCA	1									1
MEXICO	HUIXQUILUCAN					1		4	4	2	11
MEXICO	ISIDRO FABELA						2				2

MEXICO	IXTAPALUCA	1	6	2	2		10	1	5	14	41
MEXICO	IXTLAHUACA									1	1
MEXICO	JALTENCO				2						2
MEXICO	JILOTEPEC		1					1	2		4
MEXICO	JILOTZINGO				1						1
MEXICO	LA PAZ	2					4	2		2	10
MEXICO	NAUCALPAN DE JUAREZ	3	6	7	9	6	5	7	1	4	48
MEXICO	NEXTLALPAN				2						2
MEXICO	NEZAHUALCOYOTL	15	11	4	4	6	10	6	7	2	65
MEXICO	NICOLAS ROMERO		1		2		5		1	4	13
MEXICO	NOPALTEPEC				3						3
MEXICO	OCOYOACAC		2								2
MEXICO	OTUMBA								1		1
MEXICO	OZUMBA						1				1
MEXICO	SAN SIMON DE GUERRERO								1		1
MEXICO	SOYANIQUILPAN DE JUAREZ							1			1
MEXICO	SULTEPEC							2	1	2	5
MEXICO	TECAMAC				2		2			1	5
MEXICO	TEJUPILCO								1		1
MEXICO	TEMOAYA							2	3		5
MEXICO	TENANCINGO	1	1								2
MEXICO	TEOLOYUCAN			2	1						3
MEXICO	TEOTIHUACAN								7		7
MEXICO	TEPOTZOTLAN			1				1			2
MEXICO	TEQUIXQUIAC									1	1
MEXICO	TEXCOCO	1			1		2	1	2		7
MEXICO	TEZOYUCA						1				1
MEXICO	TLALMANALCO		5	5	3					1	14
MEXICO	TLALNEPANTLA DE BAZ		3		3	2	3	1	3	14	29
MEXICO	TOLUCA	4	2	2		1	1	2		1	13
MEXICO	TULTEPEC						1		1		2
MEXICO	TULTITLAN			2		1	1	1		3	8
MEXICO	VALLE DE BRAVO	1			3		1		2	1	8
MEXICO	VALLE DE CHALCO SOLIDARIDAD	1	1	1	2	4		15	1		25
MEXICO	VILLA VICTORIA			1							1
MEXICO	ZACUALPAN								1		1
MEXICO	ZINACANTEPEC			1							1
MEXICO	ZUMPAHUACAN						1				1
MEXICO	ZUMPANGO			1	2		1	3			7
MICHOACAN	AGUILILLA		1			1					2
MICHOACAN	APATZINGAN	6		15	3	6	4	9	2	1	46
MICHOACAN	APORO	1									1
MICHOACAN	AQUILA	1	1	2		7			1		12
MICHOACAN	ARIO		1						1	1	3
MICHOACAN	ARTEAGA		1							1	2
MICHOACAN	BRISEÑAS			2	1	2	3		1		9
MICHOACAN	BUENAVISTA							2		1	3
MICHOACAN	CARACUARO							1	4	1	6
MICHOACAN	CHARO			1	2		1			1	5
MICHOACAN	CHERAN					1	1				2
MICHOACAN	CHINICUILA							3			3
MICHOACAN	CHURINTZIO									1	1
MICHOACAN	CHURUMUCO				3			3		1	7

MICHOACAN	COAHUAYANA				1				2	4	7
MICHOACAN	COALCOMAN DE VAZQUEZ PALLARES			1				2	5		8
MICHOACAN	COENEO								1		1
MICHOACAN	COJUMATLAN DE REGULES				8	1	2				11
MICHOACAN	COPANDARO				1						1
MICHOACAN	CUITZEO								1		1
MICHOACAN	ECUANDUREO	2	2								4
MICHOACAN	GABRIEL ZAMORA		1				1				2
MICHOACAN	HIDALGO		1				2		7	9	19
MICHOACAN	HUETAMO		1	1	1				1	1	5
MICHOACAN	IXTLAN					1	1		4		6
MICHOACAN	JACONA			1							1
MICHOACAN	JIQUILPAN			1	2	1	4			1	9
MICHOACAN	JOSE SIXTO VERDUZCO								1		1
MICHOACAN	JUAREZ							4		2	6
MICHOACAN	JUNGAPEO	1									1
MICHOACAN	LA HUACANA	1									1
MICHOACAN	LA PIEDAD	2	2	1		1		3			9
MICHOACAN	LAGUNILLAS		1	1						2	4
MICHOACAN	LAZARO CARDENAS	4	3	5	2	3	12	5	3	1	38
MICHOACAN	LOS REYES								2	1	3
MICHOACAN	MARCOS CASTELLANOS									2	2
MICHOACAN	MORELIA	6	12	9	11	10	22	6	9	6	91
MICHOACAN	MUGICA			1		1				1	3
MICHOACAN	NAHUATZEN	1									1
MICHOACAN	NOCUPETARO								1		1
MICHOACAN	NUEVO PARANGARICUTIRO			1							1
MICHOACAN	NUEVO URECHO						3				3
MICHOACAN	PAJACUARAN	2									2
MICHOACAN	PARACHO				1		8	2	2		13
MICHOACAN	PARACUARO			1		1			1		3
MICHOACAN	PATZCUARO	1		2	1	2			1		7
MICHOACAN	PUREPERO						1				1
MICHOACAN	PURUANDIRO								2		2
MICHOACAN	QUERENDARO					2					2
MICHOACAN	SAHUAYO						2				2
MICHOACAN	SALVADOR ESCALANTE								2		2
MICHOACAN	SAN LUCAS	1		1		1					3
MICHOACAN	SUSUPUATO									2	2
MICHOACAN	TACAMBARO	1				5	1		9		16
MICHOACAN	TANCITARO	1				2			1		4
MICHOACAN	TANGAMANDAPIO		2	1							3
MICHOACAN	TANHUATO				2	1		1	1	1	6
MICHOACAN	TARETAN	1				1					2
MICHOACAN	TARIMBARO	2	1				6	3	1		13
MICHOACAN	TEPALCATEPEC							4	1		5
MICHOACAN	TINGAMBATO				1						1
MICHOACAN	TINGÜINDIN								1		1
MICHOACAN	TIQUICHEO DE NICOLAS ROMERO			2		1	2	1	1	3	10
MICHOACAN	TLALPUJAHUA				1						1
MICHOACAN	TOCUMBO	2									2
MICHOACAN	TUMBISCATIO							2			2
MICHOACAN	TUZANTLA			1		1			1		3

MICHOACAN	TZITZIO			1		1		2			4
MICHOACAN	URUAPAN	1	2	2	2	3	1	9		3	23
MICHOACAN	VENUSTIANO CARRANZA			5	1					1	7
MICHOACAN	VILLAMAR						1				1
MICHOACAN	VISTA HERMOSA	1	3		2	1					7
MICHOACAN	YURECUARO			3	1	9	7	5	3	1	29
MICHOACAN	ZACAPU							1			1
MICHOACAN	ZAMORA	2	4	2	4					1	13
MICHOACAN	ZIRACUARETIRO	1									1
MICHOACAN	ZITACUARO	1	4			1	1		1		8
MORELOS	AXOCHIAPAN								1		1
MORELOS	AYALA	1		1							2
MORELOS	CUAUTLA	2	5		4		1		3	2	17
MORELOS	CUERNAVACA	6	9	7	5	3	4	4	9	5	52
MORELOS	EMILIANO ZAPATA	3		2	5	2	6	1	5	1	25
MORELOS	HUITZILAC	1		2			1	1		1	6
MORELOS	JIUTEPEC	1		1	2	1		1			6
MORELOS	JOJUTLA	1	2	1	2	1		1	1	1	10
MORELOS	MIACATLAN					2	2	2		1	7
MORELOS	PUENTE DE IXTLA		2	2		2	1	2	2	2	13
MORELOS	TEMIXCO	2	3	10	1	3		2	5	2	28
MORELOS	TEPALCINGO	1									1
MORELOS	TEPOZTLAN				1			2			3
MORELOS	TETECALA					1					1
MORELOS	TLALTIZAPAN			1		1	1		1		4
MORELOS	TLAQUILTENANGO									1	1
MORELOS	TLAYACAPAN				1						1
MORELOS	XOCHITEPEC		1	4	2	1		1	1	4	14
MORELOS	YAUTEPEC		1	2	2	1		1	1	1	9
MORELOS	YECAPIXTLA					1				1	2
MORELOS	ZACATEPEC	1						1			2
MORELOS	ZACUALPAN	1									1
NAYARIT	ACAPONETA			1	4	5	1		1	2	14
NAYARIT	AHUACATLAN					1					1
NAYARIT	AMATLAN DE CAÑAS							1			1
NAYARIT	COMPOSTELA							1			1
NAYARIT	HUAJICORI	1				2	1	1			5
NAYARIT	IXTLAN DEL RIO							6			6
NAYARIT	LA YESCA									1	1
NAYARIT	ROSAMORADA	3		1		1					5
NAYARIT	RUIZ					32	3		2	4	41
NAYARIT	SAN BLAS				1	1		1		1	4
NAYARIT	SANTA MARIA DEL ORO	3						1	1		5
NAYARIT	SANTIAGO IXCUINTLA		5			2		2	1	2	12
NAYARIT	TECUALA	1		2	6	2	2	1	3		17
NAYARIT	TEPIC	26	19	38	28	10	18	14	33	10	196
NAYARIT	TUXPAN		6			2	6				14
NAYARIT	XALISCO	6		3	6	3	1	8	1	2	30
NUEVO LEON	ABASOLO									1	1
NUEVO LEON	AGUALEGUAS								7		7
NUEVO LEON	ALLENDE	3	3	2		1	2	4	7		22
NUEVO LEON	ANAHUAC						1	4			5
NUEVO LEON	APODACA	5	2	7	1	26	18	13	8	9	89

NUEVO LEON	CADEREYTA JIMENEZ	3	5		15	5	5	6	11	14	64
NUEVO LEON	CARMEN	1						2		1	4
NUEVO LEON	CHINA	3	1	6					4		14
NUEVO LEON	CIENEGA DE FLORES					1				5	6
NUEVO LEON	DOCTOR ARROYO							3			3
NUEVO LEON	ESCOBEDO									1	1
NUEVO LEON	GALEANA	1									1
NUEVO LEON	GARCIA	1	2	1			3				7
NUEVO LEON	GRAL. ESCOBEDO	3		7	3	2	3	5	1	4	28
NUEVO LEON	GRAL. TERAN	4	2		3		7		2		18
NUEVO LEON	GRAL. TREVIÑO					2					2
NUEVO LEON	GRAL. ZUAZUA	2		1							3
NUEVO LEON	GUADALUPE	7	18	9	9	32	18	19	6	13	131
NUEVO LEON	HUALAHUISES						3				3
NUEVO LEON	JUAREZ		5	2	8	10	8	18	2	1	54
NUEVO LEON	LINARES	3		6	3	10		3		1	26
NUEVO LEON	LOS ALDAMAS				6						6
NUEVO LEON	LOS HERRERAS			1							1
NUEVO LEON	LOS RAMONES			3			2			2	7
NUEVO LEON	MARIN							4			4
NUEVO LEON	MONTEMORELOS	5		3	1	6	2	3	3		23
NUEVO LEON	MONTERREY	46	24	19	24	36	55	78	76	41	399
NUEVO LEON	PESQUERIA	3	1				4	4	3		15
NUEVO LEON	SABINAS HIDALGO	1	2				1	7		1	12
NUEVO LEON	SALINAS VICTORIA	2				1	5				8
NUEVO LEON	SAN NICOLAS DE LOS GARZA	7	8	14	7	7	8	10	9	7	77
NUEVO LEON	SAN PEDRO GARZA GARCIA	1	4			1		3			9
NUEVO LEON	SANTA CATARINA	5	6		8	1	4	11	1	2	38
NUEVO LEON	SANTIAGO	4	1		2		4	15	10	9	45
OAXACA	ACATLAN DE PEREZ FIGUEROA									1	1
OAXACA	ASUNCION IXTALTEPEC						1				1
OAXACA	HEROICA CIUDAD DE HUAJUAPAN DE LEON	1									1
OAXACA	HEROICA CIUDAD DE JUCHITAN DE ZARAGOZA	1				1			1		3
OAXACA	LOMA BONITA		2		1	2			2	2	9
OAXACA	MESONES HIDALGO				1						1
OAXACA	MIAHUATLAN DE PORFIRIO DIAZ				1				1		2
OAXACA	OAXACA DE JUAREZ	2		1						1	4
OAXACA	OCOTLAN DE MORELOS	1									1
OAXACA	PINOTEPA DE DON LUIS								2		2
OAXACA	PUTLA VILLA DE GUERRERO	1					3		1		5
OAXACA	SALINA CRUZ	3		2							5
OAXACA	SAN AGUSTIN CHAYUCO				1						1
OAXACA	SAN AGUSTIN LOXICHA					1					1
OAXACA	SAN AGUSTIN YATARENI	1									1
OAXACA	SAN ANDRES HUAXPALTEPEC		1								1
OAXACA	SAN ANTONIO DE LA CAL								1	1	2
OAXACA	SAN JACINTO AMILPAS									3	3
OAXACA	SAN JUAN BAUTISTA GUELACHE								1		1
OAXACA	SAN JUAN BAUTISTA JAYACATLAN	1									1
OAXACA	SAN JUAN BAUTISTA TUXTEPEC	2	4		5	3		3	1	1	19
OAXACA	SAN JUAN DEL ESTADO								2		2
OAXACA	SAN JUAN LACHAO	1									1
OAXACA	SAN JUAN MIXTEPEC									1	1

OAXACA	SAN JUAN QUIAHIJE					2					2
OAXACA	SAN MIGUEL SOYALTEPEC								1		1
OAXACA	SAN PEDRO IXTLAHUACA								1		1
OAXACA	SAN PEDRO MIXTEPEC							2			2
OAXACA	SAN PEDRO POCHUTLA							1			1
OAXACA	SAN PEDRO TAPANATEPEC									4	4
OAXACA	SAN PEDRO TOTOLAPA		1				1				2
OAXACA	SAN SEBASTIAN TECOMAXTLAHUACA					1					1
OAXACA	SAN SEBASTIAN TUTLA	1									1
OAXACA	SANTA CRUZ XOXOCOTLAN							1			1
OAXACA	SANTA LUCIA DEL CAMINO	2									2
OAXACA	SANTA MARIA CORTIJO	1									1
OAXACA	SANTA MARIA HUATULCO									1	1
OAXACA	SANTA MARIA TONAMECA							1			1
OAXACA	SANTA MARIA ZACATEPEC				2			1			3
OAXACA	SANTIAGO JAMILTEPEC	1		1	1	1					4
OAXACA	SANTIAGO LAOLLAGA									3	3
OAXACA	SANTIAGO PINOTEPA NACIONAL			5							5
OAXACA	SANTIAGO TETEPEC	1									1
OAXACA	SANTO DOMINGO TEHUANTEPEC							1			1
OAXACA	TLACOLULA DE MATAMOROS				1						1
OAXACA	TLALIXTAC DE CABRERA		1								1
OAXACA	TRINIDAD ZAACHILA						1				1
PUEBLA	ACATLAN			1							1
PUEBLA	ATZITZINTLA			1							1
PUEBLA	CALPAN		1	1							2
PUEBLA	CHALCHICOMULA DE SESMA		1								1
PUEBLA	CHIETLA			2							2
PUEBLA	CHIGNAHUAPAN		3				1				4
PUEBLA	COHUECAN								1		1
PUEBLA	COXCATLAN								1		1
PUEBLA	CUAUTLANCINGO					1					1
PUEBLA	GUADALUPE			1							1
PUEBLA	IZUCAR DE MATAMOROS		3								3
PUEBLA	JUAN GALINDO							2			2
PUEBLA	MOLCAXAC			1							1
PUEBLA	NICOLAS BRAVO								1		1
PUEBLA	NOPALUCAN					1					1
PUEBLA	ORIENTAL						1				1
PUEBLA	PUEBLA	1	1		2		1		2		7
PUEBLA	SAN ANDRES CHOLULA		1			1					2
PUEBLA	SAN MARTIN TEXMELUCAN						2				2
PUEBLA	SAN PEDRO CHOLULA	1									1
PUEBLA	TECAMACHALCO			1							1
PUEBLA	TEHUACAN				1	2					3
PUEBLA	TEPEOJUMA	1									1
PUEBLA	TEZIUTLAN								2		2
PUEBLA	TILAPA						2				2
PUEBLA	TLAHUAPAN				1						1
PUEBLA	VENUSTIANO CARRANZA		1								1
PUEBLA	XICOTEPEC								2		2
PUEBLA	XOCHILTEPEC					1					1
PUEBLA	ZACAPALA			3							3

PUEBLA	ZACATLAN		1							1	2
QUERETARO	AMEALCO DE BONFIL		5								5
QUERETARO	EL MARQUES			2							2
QUERETARO	HUIMILPAN			2							2
QUERETARO	JALPAN DE SERRA									1	1
QUERETARO	PEDRO ESCOBEDO								2		2
QUERETARO	QUERETARO				2	2				1	5
QUINTANA ROO	BENITO JUAREZ	5	1	7	1	5	6	3	5	1	34
QUINTANA ROO	FELIPE CARRILLO PUERTO									1	1
QUINTANA ROO	ISLA MUJERES		1							1	2
QUINTANA ROO	OTHON P. BLANCO		1	1					1	2	5
QUINTANA ROO	SOLIDARIDAD	2							2	1	5
QUINTANA ROO	TULUM									1	1
SAN LUIS POTOSI	CEDRAL	2									2
SAN LUIS POTOSI	CERRO DE SAN PEDRO	1		1							2
SAN LUIS POTOSI	CIUDAD VALLES		2	1	1	3			2		9
SAN LUIS POTOSI	EBANO	3	3			6					12
SAN LUIS POTOSI	GUADALCAZAR				1						1
SAN LUIS POTOSI	HUEHUETLAN					2					2
SAN LUIS POTOSI	MATEHUALA	9									9
SAN LUIS POTOSI	MOCTEZUMA						2				2
SAN LUIS POTOSI	RIOVERDE									2	2
SAN LUIS POTOSI	SAN CIRO DE ACOSTA		2				4				6
SAN LUIS POTOSI	SAN LUIS POTOSI	12	12	7	4	8		7		1	51
SAN LUIS POTOSI	SAN VICENTE TANCUAYALAB	3	1	1					1		6
SAN LUIS POTOSI	SANTA MARIA DEL RIO		1								1
SAN LUIS POTOSI	SOLEDAD DE GRACIANO SANCHEZ	4	2	3		1		1			11
SAN LUIS POTOSI	TAMASOPO									3	3
SAN LUIS POTOSI	TAMUIN		3			1				1	5
SAN LUIS POTOSI	TANCANHUITZ				2						2
SAN LUIS POTOSI	VILLA DE ARISTA					3					3
SAN LUIS POTOSI	VILLA DE ARRIAGA					1					1
SAN LUIS POTOSI	VILLA DE RAMOS					3					3
SAN LUIS POTOSI	VILLA DE REYES									1	1
SAN LUIS POTOSI	VILLA JUAREZ				3						3
SAN LUIS POTOSI	XILITLA			2							2
SINALOA	AHOME	7	15	8	19	3	13	4	8	14	91
SINALOA	ANGOSTURA	4	3	10	5			1	3	3	29
SINALOA	BADIRAGUATO	2	1	1		3	5		7	2	21
SINALOA	CHOIX	2		4	5	11	1		4	1	28
SINALOA	CONCORDIA				2	2		1			5
SINALOA	COSALA	2	1	2	1	1	1		17		25
SINALOA	CULIACAN	34	28	30	74	33	40	44	47	35	365
SINALOA	EL FUERTE	2	2	2	7	2	10	5	3	9	42
SINALOA	ELOTA	1	1				1				3
SINALOA	ESCUINAPA		1			1		1	1	2	6
SINALOA	GUASAVE	2	7	15	6	1	3	13	1	5	53
SINALOA	MAZATLAN	33	25	29	20	15	18	23	8	20	191
SINALOA	MOCORITO	6	1	3	8	5	5	8	8	2	46
SINALOA	NAVOLATO	13	6	4	16	11	6	10	12	14	92
SINALOA	ROSARIO		3	1		1		4	1		10
SINALOA	SALVADOR ALVARADO	5	3	6	11	11	8		4	4	52
SINALOA	SAN IGNACIO	1		4		4	2		8		19

SINALOA	SINALOA		1	4	2	1	4	7	2	1	22
SONORA	AGUA PRIETA	2	2	8		2	3	1			18
SONORA	ALAMOS					1	2				3
SONORA	BACUM	1									1
SONORA	BENITO JUAREZ							1		1	2
SONORA	CABORCA	5			2		2	1	1	2	13
SONORA	CAJEME	7	8	1	9	1	2	2	3	3	36
SONORA	CANANEA		2		1	2					5
SONORA	CUCURPE				2					3	5
SONORA	EMPALME	1									1
SONORA	ETCHOJOA	1			1						2
SONORA	GENERAL PLUTARCO ELIAS CALLES	4		14		1	2	1	3	1	26
SONORA	GUAYMAS				1					1	2
SONORA	HERMOSILLO	8	8	8	2	3	2	4	2	5	42
SONORA	HUATABAMPO					1	1	1		2	5
SONORA	IMURIS		1		1					2	4
SONORA	MAGDALENA							1		1	2
SONORA	NACO	1			1						2
SONORA	NAVOJOA									1	1
SONORA	NOGALES	3	3	16	8	4	1	5	1	6	47
SONORA	PITIQUITO	3									3
SONORA	PUERTO PEÑASCO	3		1	3		1			2	10
SONORA	SAN LUIS RIO COLORADO	1		3							4
SONORA	TUBUTAMA			2		1				1	4
SONORA	YECORA	1									1
TABASCO	BALANCAN							1			1
TABASCO	CARDENAS	3	2	6	3	10	3	6	4		37
TABASCO	CENTRO	4		1			1	4	1	1	12
TABASCO	COMALCALCO					2		1			3
TABASCO	CUNDUACAN			2		3	3			2	10
TABASCO	HUIMANGUILLO		1	4	1		3			1	10
TABASCO	JALPA DE MENDEZ						2				2
TABASCO	JONUTA								2		2
TABASCO	MACUSPANA				1						1
TABASCO	NACAJUCA					1					1
TABASCO	TACOTALPA			1							1
TABASCO	TENOSIQUE				1	3	1		3		8
TAMAULIPAS	ABASOLO			20							20
TAMAULIPAS	ALDAMA						4				4
TAMAULIPAS	ALTAMIRA	2	1	3	4	6	4		5		25
TAMAULIPAS	CAMARGO	1				4					5
TAMAULIPAS	CASAS	4			3					1	8
TAMAULIPAS	CIUDAD MADERO			1	2	5		1		9	18
TAMAULIPAS	EL MANTE		1		5	4		3	1		14
TAMAULIPAS	GONZALEZ	1		3	1	6	2	1	5		19
TAMAULIPAS	GÜEMEZ					2	1	1			4
TAMAULIPAS	GUERRERO	4	3	3		13	2	5		5	35
TAMAULIPAS	GUSTAVO DIAZ ORDAZ	5				6					11
TAMAULIPAS	HIDALGO	7	1	3	2	17	1	10	1	4	46
TAMAULIPAS	JIMENEZ	2			2		1				5
TAMAULIPAS	LLERA	1			1		1		1		4
TAMAULIPAS	MAINERO							3			3
TAMAULIPAS	MATAMOROS	3	2	6	5	7	7	14	7	21	72

TAMAULIPAS	MIER	3	3	3	14	8	10		5	4	50
TAMAULIPAS	MIGUEL ALEMAN		10		6	5	2				23
TAMAULIPAS	NUEVO LAREDO	4	31	22	24	6	2	9	29	17	144
TAMAULIPAS	PADILLA	1	18						1	2	22
TAMAULIPAS	REYNOSA	6		1	11	6	10	4	7	6	51
TAMAULIPAS	RIO BRAVO	3		9			1			1	14
TAMAULIPAS	SAN FERNANDO	6	11	24	200	24	11	3	12	1	292
TAMAULIPAS	SOTO LA MARINA		3	3				1			7
TAMAULIPAS	TAMPICO	9	1	11	7	11	9	9		6	63
TAMAULIPAS	VALLE HERMOSO	28	18	35		4		9	1		95
TAMAULIPAS	VICTORIA		4	14	2	6	10		4	7	47
TAMAULIPAS	VILLAGRAN			1		1			1	2	5
TAMAULIPAS	XICOTENCATL				2						2
TLAXCALA	CALPULALPAN								2		2
TLAXCALA	HUEYOTLIPAN			1							1
TLAXCALA	IXTACUIXTLA DE MARIANO MATAMOROS						2				2
TLAXCALA	TLAXCO			2							2
VERACRUZ	ACTOPAN							1			1
VERACRUZ	ACULTZINGO			2							2
VERACRUZ	ALAMO TEMAPACHE			1	1						2
VERACRUZ	ALTOTONGA		1								1
VERACRUZ	ALVARADO		5							2	7
VERACRUZ	AMATITLAN			3							3
VERACRUZ	AMATLAN DE LOS REYES								2		2
VERACRUZ	ATZALAN					1					1
VERACRUZ	BOCA DEL RIO					5	3	10	15	61	94
VERACRUZ	CARLOS A. CARRILLO				5						5
VERACRUZ	CATEMACO			3							3
VERACRUZ	CERRO AZUL								1		1
VERACRUZ	CHALMA								1		1
VERACRUZ	CHICONAMEL									1	1
VERACRUZ	COATZACOALCOS			2	1			4	3	1	11
VERACRUZ	COSAMALOAPAN DE CARPIO	1		2		1				1	5
VERACRUZ	COSCOMATEPEC			1							1
VERACRUZ	COSOLEACAQUE	1		1	4					1	7
VERACRUZ	COTAXTLA					1					1
VERACRUZ	CUITLAHUAC						1				1
VERACRUZ	EL HIGO			7	12						19
VERACRUZ	EMILIANO ZAPATA		1								1
VERACRUZ	FORTIN			1							1
VERACRUZ	HUAYACOCOTLA						2				2
VERACRUZ	HUEYAPAN DE OCAMPO	1									1
VERACRUZ	IGNACIO DE LA LLAVE							1			1
VERACRUZ	ISLA		2	2			2			1	7
VERACRUZ	IXHUATLAN DE MADERO	1			1				1		3
VERACRUZ	IXMATLAHUACAN			1							1
VERACRUZ	JALTIPAN					1			1		2
VERACRUZ	JOSE AZUETA									1	1
VERACRUZ	LA ANTIGUA				1						1
VERACRUZ	LAS CHOAPAS			2	1		1			1	5
VERACRUZ	LAS VIGAS DE RAMIREZ							1			1
VERACRUZ	MANLIO FABIO ALTAMIRANO					1					1
VERACRUZ	MARTINEZ DE LA TORRE	1		2				1	2		6

VERACRUZ	MEDELLIN		1				1	1		2	5
VERACRUZ	MINATITLAN			1							1
VERACRUZ	NANCHITAL DE LAZARO CARDENAS DEL RIO	1									1
VERACRUZ	NARANJOS AMATLAN	2									2
VERACRUZ	OLUTA									1	1
VERACRUZ	OMEALCA	1									1
VERACRUZ	OTATITLAN					2			1		3
VERACRUZ	OZULUAMA DE MASCAREÑAS						1				1
VERACRUZ	PANUCO	9	9	1		2	19	11	1	2	54
VERACRUZ	PAPANTLA						2				2
VERACRUZ	PASO DE OVEJAS				1						1
VERACRUZ	PLAYA VICENTE					3	1		2		6
VERACRUZ	POZA RICA DE HIDALGO	1		1							2
VERACRUZ	PUEBLO VIEJO	3					9	6	2	2	22
VERACRUZ	PUENTE NACIONAL		1								1
VERACRUZ	SAN ANDRES TUXTLA								1		1
VERACRUZ	SAN JUAN EVANGELISTA	1						2			3
VERACRUZ	SANTIAGO TUXTLA			1							1
VERACRUZ	SAYULA DE ALEMAN						1				1
VERACRUZ	TANCOCO		1								1
VERACRUZ	TANTIMA								3		3
VERACRUZ	TANTOYUCA			1	1	1	1	1	1		6
VERACRUZ	TEMPOAL				1						1
VERACRUZ	TEXCATEPEC					1					1
VERACRUZ	TIERRA BLANCA		2	1	1			1		3	8
VERACRUZ	TLALIXCOYAN									1	1
VERACRUZ	TRES VALLES			2	3					1	6
VERACRUZ	TUXPAN	2					1		2	2	7
VERACRUZ	VERACRUZ		2	5	6		19	21	57	45	155
VERACRUZ	XALAPA	14				1	12	2		6	35
VERACRUZ	YANGA						1				1
VERACRUZ	ZONTECOMATLAN DE LOPEZ Y FUENTES					1					1
YUCATAN	TIZIMIN						1				1
ZACATECAS	BENITO JUAREZ					13			1		14
ZACATECAS	FRESNILLO							15			15
ZACATECAS	GENERAL FRANCISCO R. MURGUIA		2								2
ZACATECAS	GUADALUPE			1	1						2
ZACATECAS	JALPA	3		1					11		15
ZACATECAS	JEREZ						2	1		3	6
ZACATECAS	JUAN ALDAMA									3	3
ZACATECAS	MONTE ESCOBEDO							1			1
ZACATECAS	NOCHISTLAN DE MEJIA				3						3
ZACATECAS	NORIA DE ANGELES									2	2
ZACATECAS	OJOCALIENTE									2	2
ZACATECAS	PANUCO						2				2
ZACATECAS	RIO GRANDE		1	2							3
ZACATECAS	SOMBRERETE	3		8					1		12
ZACATECAS	TABASCO		9								9
ZACATECAS	TEPECHITLAN	1									1
ZACATECAS	TEPETONGO		5								5
ZACATECAS	TEUL DE GONZALEZ ORTEGA							10			10
ZACATECAS	TRANCOSO							1			1
ZACATECAS	TRINIDAD GARCIA DE LA CADENA									3	3

ZACATECAS	VALPARAISO	3									3
ZACATECAS	ZACATECAS		3		2					9	14
TOTAL		1,351	1,176	1,424	1,630	1,539	1,433	1,519	1,461	1,370	12,903
